# Nonlinear Dynamics in Gene Regulation Promote Robustness and Evolvability of Gene Expression Levels

**DOI:** 10.1371/journal.pone.0153295

**Published:** 2016-04-15

**Authors:** Arno Steinacher, Declan G. Bates, Ozgur E. Akman, Orkun S. Soyer

**Affiliations:** 1 MRC Biostatistics Unit, Cambridge, United Kingdom; 2 School of Engineering, University of Warwick, Warwick, United Kingdom; 3 College of Engineering, Mathematics, and Physical Sciences, University of Exeter, Exeter, United Kingdom; 4 School of Life Sciences, University of Warwick, Warwick, United Kingdom; UC Santa Barbara, UNITED STATES

## Abstract

Cellular phenotypes underpinned by regulatory networks need to respond to evolutionary pressures to allow adaptation, but at the same time be robust to perturbations. This creates a conflict in which mutations affecting regulatory networks must both generate variance but also be tolerated at the phenotype level. Here, we perform mathematical analyses and simulations of regulatory networks to better understand the potential trade-off between robustness and evolvability. Examining the phenotypic effects of mutations, we find an inverse correlation between robustness and evolvability that breaks only with nonlinearity in the network dynamics, through the creation of regions presenting sudden changes in phenotype with small changes in genotype. For genotypes embedding low levels of nonlinearity, robustness and evolvability correlate negatively and almost perfectly. By contrast, genotypes embedding nonlinear dynamics allow expression levels to be robust to small perturbations, while generating high diversity (evolvability) under larger perturbations. Thus, nonlinearity breaks the robustness-evolvability trade-off in gene expression levels by allowing disparate responses to different mutations. Using analytical derivations of robustness and system sensitivity, we show that these findings extend to a large class of gene regulatory network architectures and also hold for experimentally observed parameter regimes. Further, the effect of nonlinearity on the robustness-evolvability trade-off is ensured as long as key parameters of the system display specific relations irrespective of their absolute values. We find that within this parameter regime genotypes display low and noisy expression levels. Examining the phenotypic effects of mutations, we find an inverse correlation between robustness and evolvability that breaks only with nonlinearity in the network dynamics. Our results provide a possible solution to the robustness-evolvability trade-off, suggest an explanation for the ubiquity of nonlinear dynamics in gene expression networks, and generate useful guidelines for the design of synthetic gene circuits.

## Introduction

Biological systems are subject to random mutations as well as noise arising from internal and external stochastic fluctuations. It is important that the potential perturbing effects of noise and mutation are buffered at the phenotype level, i.e. biological systems are expected to display a phenotype that is robust to these perturbations [1–3]. However, changes in fitness pressures over evolutionary time make it similarly important that biological systems are able to produce heritable phenotypic variants that are adaptive. This ability has often been termed evolvability [4–6]. How can biological systems generate phenotypes that are robust to mutations and noise, yet also able to evolve through the effects of these same perturbations [7]?

A suggested solution to this trade-off is that robustness of a phenotype to mutations could allow the accumulation of genotypic diversity, which could then translate into phenotypic diversity under subsequent mutations or changes in selective pressures [8]. In support of this idea, analysis of computational models of diverse biological systems has shown that there exist mutationally linked genotypes that display the same phenotype, forming a so-called neutral network [9–11], and that these genotypes can still have access to high phenotypic diversity [8, 12–14]. It has also been found that the size of accessible neutral neighborhood for genotypes determines speed of adaptation [15]. Confirming these theoretical findings, experiments on several biological systems have found these systems to display phenotypes that are robust to most mutations [1, 16, 17], but at the same time able to display high levels of phenotypic diversity under certain mutations [6, 16, 18]. Furthermore, it is shown experimentally that a period of neutral evolution of RNA enzymes under one selective pressure increased the speed of evolution under a different selection pressure [19].

While these findings suggest that robustness can increase evolvability in a population context, they do not provide any mechanistic understanding of how the potential evolvability-robustness trade-off can be broken at the level of genotypes. A clearer understanding of the evolvability-robustness relationship at the genotype level requires the formulation of definitions of robustness and evolvability that permit their quantification across a large class of genotypes and mutations. So far, most computational studies have used a discretized set of mutations to measure the robustness of genotypes and the phenotypic diversity available to them [9, 10, 14, 20–23]. When the phenotypic diversity is used as a proximate measure for evolvability, these studies have found an inverse correlation between robustness and evolvability [21–24]. It is not clear, however, how this result depends on the discretisation method used. In particular, a number of studies indicate that genotypes’ robustness to noise (i.e. intrinsic variation of gene expresssion levels related to infinitesimally small perturbations of system parameters) and mutation (i.e. large perturbations of system parameters, affecting gene expression variability) are interlinked with their evolvability [25–27]. Other studies have used *in silico* evolution, and measured evolvability as the change in evolutionary fitness with respect to mutation size [24, 28–30] or as the speed or frequency of the emergence of specific phenotypes [9, 31]. Some of these studies have indicated a positive relation between robustness and evolvability [31], but such conclusions are likely to be dependent on the choice of fitness function, and other details of the *in silico* simulations.

In order to overcome these limitations and provide a comprehensive analysis of the relationship between robustness and evolvability at the genotype level, here we develop several mathematically rigorous measures for robustness and evolvability, and apply these to genotypes defined for two common gene regulation network architectures involving a single gene under self-regulation or under the regulation of an upstream transcription factor. Evaluating several million genotypes for each of these systems and using the developed measures, we show that increased nonlinearity in the system dynamics breaks the inverse correlation between robustness and evolvability. We analyze the genotype-phenotype mapping in these circuits by varying the degree of nonlinearity in the equations governing gene expression (see Methods). Thus, we distinguish between genotypes encoding for “linear” and “nonlinear” dynamics and their resulting phenotypes as steady state levels of gene expression. Our finding holds irrespective of the mutational distributions considered for measuring these quantities. We find that robust and evolvable genotypes display low expression levels and occupy a special region, presenting sudden changes in phenotype with small changes in genotype. These findings suggest that nonlinear system dynamics in gene regulation are crucial for maintaining robustness and evolvability of expression levels. Furthermore, they predict that the empirically found correlation between gene expression noise and plasticity [25, 32] results from nonlinearity in gene regulatory systems.

## Material and Methods

### Gene circuit models

To study robustness and evolvability in the context of gene regulatory networks, we consider here two network architectures that are commonly observed in nature. This analysis considers the system dynamics of these networks in isolation from other cellular components. The two circuits we consider are described in detail in the following sections.

#### Circuit I—Auto-activation model

In this network architecture, it is assumed that translated protein positively regulates the transcription of its own gene by binding to its cis-regulatory module. Such regulation is common in biology; moreover, synthetic implementations of auto-activation feedback motifs have demonstrated experimentally that they can give rise to bistable system dynamics in which two distinct steady state expression levels are possible [33, 34]. The particular model of auto-activation feedback that we considered comprises four reaction processes: transcription, translation, mRNA degradation and protein degradation.

The equations governing the time evolution of the concentrations of mRNA (denoted *M*) and protein (denoted *P*) are shown below:

$$\begin{array}{c} \begin{array}{cc} \frac{d\left[M\right]}{dt} & =\frac{a\left(k_{1}+k_{2}{\left(\frac{\left[P\right]}{k_{D}}\right)}^{N}\right)}{1+{\left(\frac{\left[P\right]}{k_{D}}\right)}^{N}}-k_{4}\left[M\right],\\ \frac{d\left[P\right]}{dt} & =k_{3}\left[M\right]-k_{5}\left[P\right]. \end{array} \end{array}$$ (1)

Here, transcription is controlled by the maximum transcription rate *a*, the basal transcription rate *k*_1_, and the rate of feedback-mediated transcription *k*_2_. The parameter *k*_3_ denotes the translation rate, while *k*_4_ and *k*_5_ are the mRNA and protein degradation rates respectively. Finally, the Hill coefficient *N* indicates the degree of cooperativity in the feedback loop and *k*_*D*_ quantifies the protein concentration at which activation is half maximal. It is straightforward to show that nondimensionalising the steady state protein level $\bar{P}$ via

$$\begin{array}{c} X=\frac{\bar{P}}{k_{D}} \end{array}$$ (2)

leads to the following simple equation for steady state expression:

$$\begin{array}{c} f\left(X\right)=\alpha X. \end{array}$$ (3)

In the above, the function f:R→R is defined by

$$\begin{array}{c} f\left(x\right)=\frac{1+Kx^{N}}{1+x^{N}}, \end{array}$$ (4)

where

$$\begin{array}{c} \alpha =\frac{k_{D}k_{4}k_{5}}{ak_{1}k_{3}},\;K=\frac{k_{2}}{k_{1}}. \end{array}$$ (5)

It follows that steady state expression is determined by the composite parameters *α* and *K*, together with the level of nonlinearity *N*. A detailed stability analysis of the model can be found in the supplementary information (S1 File), where it is shown that the circuit can exhibit both monostability and bistability, depending on the values of *α*, *K* and *N*.

#### Circuit II—Simple-activation model

**Fig 1:**
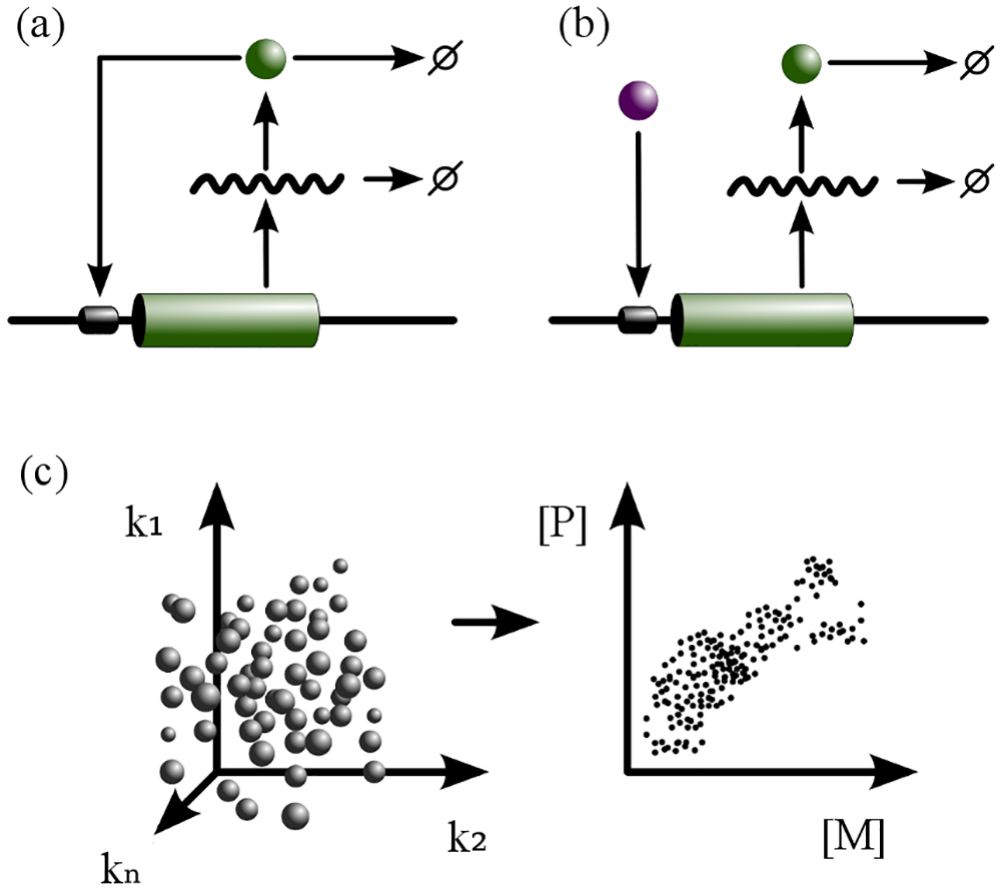
Genotype-phenotype mapping of gene regulation networks. (a): The auto-activation circuit (circuit I) consists of a single gene, whose protein product (*P*) binds the promoter region to activate the expression of its own mRNA (*M*). The sink sign indicates mRNA and protein degradation/dilution. (b): A simple-activation circuit (circuit II) in which a transcription factor (purple) binds to the promoter region of a gene to activate the expression of mRNA (*M*). The protein product (*P*) is produced by translation of *M*. (c): A schematic representation of the G-P mapping. Genotypes are represented as discrete entities in the multi-dimensional parameter space of kinetic rates (indicated by the axes *k*_1_ → *k*_*n*_) and are mapped to their corresponding phenotypes, which are steady state solutions in the two-dimensional space spanned by *M* and *P* expression levels.

In this network, gene expression is driven by an external transcription factor (TF) which is assumed to be at steady state (a schematic diagram of this circuit is shown in Fig 1b). This TF could represent, for example, the final component of a signalling pathway, such as the MAPK cascade ([35]). The model equations are

$$\begin{array}{c} \begin{array}{cc} \frac{d\left[M\right]}{dt} & =a\left(\frac{k_{1}+k_{2}{\left(\frac{T}{k_{D}}\right)}^{N}}{1+{\left(\frac{T}{k_{D}}\right)}^{N}}\right)-k_{4}\left[M\right],\\ \frac{d\left[P\right]}{dt} & =k_{3}\left[M\right]-k_{5}\left[P\right], \end{array} \end{array}$$ (6)

where mRNA and protein are represented by *M* and *P* respectively, *T* denotes the concentration of the TF, and all other parameters represent the same processes that they did previously for the auto-activation circuit in Eq (1). Gene activation is again modeled using a Hill-type function in which the Hill coefficient *N* determines the nonlinearity of the signal response.

At steady state, the protein level is determined by

$$\begin{array}{c} \bar{P}=\frac{ak_{1}k_{3}}{k_{4}k_{5}}\left(\frac{1+\frac{k_{2}}{k_{1}}{\left(\frac{T}{k_{D}}\right)}^{N}}{1+{\left(\frac{T}{k_{D}}\right)}^{N}}\right), \end{array}$$ (7)

while the corresponding steady state mRNA level is

$$\begin{array}{c} \bar{M}=\frac{k_{5}}{k_{3}}\bar{P}. \end{array}$$ (8)

There is therefore always only one steady state of the system $\left(\bar{M},\bar{P}\right)$, and it can be shown that this is always stable (see S1 File). For simplicity, we introduce the following composite parameters (cf. Eq (5)):

$$\begin{array}{c} \beta =\frac{k_{4}k_{5}}{ak_{1}k_{3}},\;K=\frac{k_{2}}{k_{1}},\;\gamma =\frac{T}{k_{D}}. \end{array}$$ (9)

Note that *γ* represents the nondimensionalized TF concentration. Also, as in the analysis of the auto-activation circuit, *K* is the ratio of the TF-mediated and basal transcription rates. The steady state protein level in this case can thus be written in the form

$$\begin{array}{c} \bar{P}=\frac{1}{\beta }f\left(\gamma \right), \end{array}$$ (10)

where *f*(*γ*) is the function defined previously in Eq (4), and is thus a function of *β*, *K*, *N* and *γ*.

#### ‘Degrees’ of nonlinearity

Both gene regulation networks analyzed here are characterized by nonlinear response dynamics. Our analysis provides arguments for the extent to which nonlinearity shapes the robustness-evolvability relationship. Since such statements could easily be interpreted in different ways, we feel the need to explain in more detail what we mean by ‘nonlinearity’ in the context of this study. In both gene regulation networks at hand, expression of mRNA (*M*) is dependent on levels of transcription factor (*P* or *T*, respectively, for circuits I and II), to the power of *N*. The parameter *N* is the Hill coefficient in the kinetic equation, directly relating to the level of cooperativity in transcriptional regulation.

From a mathematical viewpoint, expression of *M* is always nonlinear unless *N* = 0. It is well known that nonlinear response dynamics can result in ultrasensitive relationships, characterized by a sigmoidal function between signal and response [36, 37]. A system exhibiting nonlinearity leading to ultrasensitivity is characterized by insensitivity of the system response to a stimulus of a certain range of concentration, whereas outside this range the system response might be dramatic. Ultrasensitivity is one specific manifestation of nonlinear response, which can in more general terms be described as a deviation of system response towards stimuli from a perfect straight line [38]. It is this notion of nonlinearity that we refer to when we characterize our system parameter *N* in its ability to increase the nonlinearity in its regulatory response—it increases the deviation of *M* expression as a response to increase in transcription factor levels from a straight line, as quantified by the nonlinearity measure *L* in [38].

Since the regulatory networks featured in this study are specifically showing ultrasensitive response dynamics at high levels of *N*, the quantification of response coefficient *R* in [37] might be an even more relevant equivalent to our notion of increasing or decreasing levels of nonlinearity, captured by levels of *N*.

### Genotype-phenotype mappings

For both circuits, the genotype-phenotype mapping was constructed by discretizing the parameter space, and then calculating the steady state expression level of the system (phenotype) for each possible parameter combination (genotype) (Fig 1c). For circuit I, over 10 million genotypes were simulated. A large subset of these, represented by 7.77 × 10^6^ genotypes were tested based upon parameters in [29]. Of these genotypes, 6.28 × 10^6^ mapped to monostable phenotypes and 1.48 × 10^6^ mapped to bistable phenotypes. Another subset of 3.38 × 10^6^ genotypes were simulated, using parameter ranges based on experimental measurements of the *lac* operon in *Escherichia coli*. These yielded 2.85 × 10^6^ monostable phenotypes and 5.2 × 10^5^ bistable phenotypes.

For circuit II, 2 million genotypes were considered. Parameter values were obtained by randomly sampling within the specified bounds. For parameters *k*_1_, *k*_2_, *T* and *k*_*D*_, samples were taken from a lognormal distribution; the remaining parameters were sampled from a uniform distribution.

The parameter ranges used to construct the G-P mappings are presented in Tables A–C in S1 File.

### Quantifying robustness and evolvability

We use several sets of measures to quantify robustness and evolvability, with the overall aim to capture the effects of mutations of different sizes on expression levels. Before we characterize each measure in detail, we would like to point out that one reason to use several measures for both robustness and evolvability is to show that our outcomes are consistent, ie. not just artifacts of chosen measures. As some measures might only be valid for a certain size of mutations, the finding that genotypes which are robust to mutations of a certain size could be evolvable to mutations of a different size would not provide a conclusive answer to the question whether these genotypes are effectively both robust and evolvable. Therefore, we here present two measures each for robustness and evolvability: one which is valid for infinitesimally small changes in genotype, hence for mutations with a small effect, and another one which is valid for arbitrarily large changes in genotypes. We then provide results based upon combinations between these measures to establish which genotypes are both robust and evolvable irrespective of the size of a mutational effect.

#### Quantifying evolvability

Evolvability can be thought of as the ability to produce variation by mutation. For a gene regulation network, looking at steady state expression levels as phenotype, we can relate this to the ability of a genotype to give rise to different phenotypes, given mutations upon the genotype. Since we model gene expression network by ordinary differential equations based upon mass-action kinetics, genotypes are directly represented as reaction rates and hence parameter sets in differential equations. Hence, mutations can in this framework be represented as parameter changes in the set of equations characterizing a gene regulation network. Within this framework, we can formulate measures for evolvability that capture the variation of steady state expression levels (phenotypes) with regard to changes in the underlying system parameters (mutational effects).

#### Quantifying evolvability for small mutations

Gene expression noise is one way of accomplishing phenotypic variation, and it is due to very small fluctuations in genotypes (ie. Reaction rates). Hence, we use gene expression noise as a measure of evolvability, arising from small fluctuations.

We measured levels of intrinsic noise by applying the Linear Noise Approximation (LNA) to genotypes that met the necessary condition for the LNA to hold, namely their Lyapunov stability; this condition is met by genotypes that are monostable in circuit I, and by all genotypes in circuit II. LNA was performed using the approach of [39]. For our analyses, the noise measure was taken to be the covariance of the protein levels *P*, scaled (normalized) by the respective steady state expression level $\bar{P}$.

#### Quantifying evolvability for arbitrarily large mutations

While infinitesimally small fluctuations on the genotype level, resulting in expression noise, are able to produce variation on the phenotype level, the LNA framework for quantification of this variational effect is not suitable for larger mutations, since LNA is defined only for infinitesimally small changes in parameters.

For larger mutational effects, one way of quantifying evolvability would be to characterize how large the ‘spread’ of expression levels from mutated genotypes is, with regard to the overall size of mutation causing phenotypic changes. The coefficient of variation provides a straightforward quantification of the variation in expression levels, regardless of the expression level of the focal genotype, i.e. the genotype which is mutated. Since this measure is not bounded by size of mutational effect, we employ it for quantifying the effect of arbitrarily large mutations.

Following these considerations, we consider, for each focal genotype, a set of mutated genotypes *g*_*n*_ such that every member of this set differs by one parameter change from the focal one. Writing *P*_*n*_ for the corresponding set of phenotypes (i.e. steady state protein expression levels), evolvability *E* is defined as the coefficient of variation of this set, scaled by a normalizing factor *m*:

$$\begin{array}{c} E=\frac{\frac{\sigma \left(P_{n}\right)}{\mu \left(P_{n}\right)}}{m}. \end{array}$$ (11)

whereby *σ* and *μ* represent the standard deviation and the mean of the distributions of protein expression levels, respectively. We used two approaches to apply mutational changes: in the first approach, the set of mutated genotypes *g*_*n*_ consists of the 1-mutant neighbors of each focal genotype in the discretized parameter space of the genotype-phenotype mapping. In the second approach, different mutation sizes were considered, such that the set of mutated genotypes consists of fixed-percentage perturbations of single parameters. e.g. perturbations of ±5%, ±10% or ±20%.

For all evolvability measurements presented in this study, the factor *m* in Eq (11) was defined as the number of mutated genotypes that comprise *g*_*n*_. This number can vary between 8 and 16 in circuit I, depending on whether mutated parameters are at the boundaries of the tested parameter vectors or not. We also considered alternative definitions of *m*, such as the norm of all relative parameter changes

$$\begin{array}{c} m=\epsilon_{1}+\sqrt{\sum {\left(\frac{p-p_{n}}{\epsilon_{2}+p}\right)}^{2}}, \end{array}$$ (12)

where *p* refers to the parameter values of the focal genotype, *p*_*n*_ is the parameter value of its neighbors, and *ϵ*_1_, *ϵ*_2_ are small numbers (*ϵ*_1_ = *ϵ*_2_ = 10^−10^) which prevent potential divisions by zero. The results obtained using this measure were qualitatively equivalent.

#### Quantifying robustness

Robustness can be understood as the ability to withstand mutational effects, i.e. to sustain a phenotype amid changes on a genotype level. Considering our framework of kinetic equations describing gene regulation networks, robustness as described above would then relate to the insensitivity of the output of a dynamical system to changes in underlying parameters, reflecting mutational changes.

As in the case of evolvability, fluctuations on the genotype level, represented as parameter changes, can be of diverse magnitude and the quantification of robustness will depend on the scale of such changes. Considering further our introductory remarks in this section, to compare robustness and evolvability for a given genotype, we need to ensure that such a comparison is not thwarted by potential artifacts emanating from the usage of different scales for evolvability and robustness measures. Thus, following the above definitions of evolvability, we here present quantifications of robustness on two scales: one on the scale of infinitesimally small genotypic changes, and one to arbitrarily large mutations.

#### Quantifying robustness for small mutations

For mutations of small effect, we quantify the robustness as the inverse of the global sensitivity of the linearized dynamical system that it encodes. Hence, robustness to small mutations is represented as insensitivity to parameter changes in the underlying dynamical system. By focussing on insensitivity, we are able to capture the ability of the system to retain the phenotype of a corresponding focal genotype subject to mutational effects. For infinitesimally small changes at the genotypic level, a commonly used measure is the global sensitivity of the linearized system, and given our interest in insensitivity of this system, we use the inverse of global sensitivity as a quantification of robustness.

Following [3], we defined the robustness of a given genotype to mutations of small effect as the reciprocal 1/*S* of the sensitivity *S* of the steady state protein level $\bar{P}$ to parameter changes:

$$\begin{array}{c} S\;=\;{\left\|\frac{\partial \;\text{ln}\;\bar{P}}{\partial \;\text{ln}\;k}\right\|}_{2}^{2}. \end{array}$$ (13)

The global sensitivity *S*, given in this expression, is the extent of change in steady state protein expression level, given a change in model parameters. Here, ‖⋅‖_2_ represents the standard Euclidian 2-norm, and **k** = (*k*_*i*_) is the vector of model parameters. Given an ensemble $\left\{{\delta k}_{i}^{\left(j\right)}/k_{i}^{\left(j\right)}\right\}$ of zero-mean, independent, identically distributed scaled parameter perturbations, the variance of the corresponding scaled protein levels $\delta \bar{P}/\bar{P}$ is approximately given by $\text{var}(\delta \bar{P}/\bar{P})=S\;\text{var}(\delta k_{i}/k_{i})$. The sensitivity *S* thus quantifies the extent to which protein levels can be adjusted by small bounded fluctuations that affect biochemical reaction rates [40, 41]. The smaller the value of *S*, the smaller the relative change in $\bar{P}$ under parameter variations, and hence the greater the robustness of the circuit. Throughout all figures of our manuscript, the robustness measure was normalized to have a maximum value of 1.

Detailed derivations of *S* for the circuits considered in this study can be found in the SI. In the case of circuit I, *S* can be expressed in the form shown below:

$$\begin{array}{c} S=\frac{1}{{\left(\alpha -f^{\prime }\left(X\right)\right)}^{2}}\left(4\alpha^{2}+\frac{{\left(KX^{N}\right)}^{2}+1}{X^{2}{\left(1+X^{N}\right)}^{2}}+f^{\prime }{\left(X\right)}^{2}\left(\ln{\left(X\right)}^{2}+1\right)\right). \end{array}$$ (14)

It follows from Eqs (3) and (4) that the robustness of circuit I only depends on *α*, *K* and *N*. The sensitivity of circuit II is given by:

$$\begin{array}{c} S=4+\frac{1}{{\left(\beta \bar{P}\right)}^{2}}\left(\frac{{\left(K\gamma^{N}\right)}^{2}+1}{{\left(1+\gamma^{N}\right)}^{2}}+{\left(\gamma f^{\prime }\left(\gamma \right)\right)}^{2}\left(\ln{\left(\gamma \right)}^{2}+2\right)\right). \end{array}$$ (15)

Eqs (4) and (10) therefore imply that the robustness of circuit II only depends on *β*, *K*, *N* and *γ*.

#### Quantifying robustness for arbitrarily large mutations

To ensure robustness is not just capturing small perturbations around the steady state, but also larger perturbations, representing mutations of larger effect (for instance dramatic reduction of complex stability during protein degradation, captured by the protein degradation rate), we developed an additional measure that does not depend on linearization of the system, but takes a more general approach of how close expression levels of a mutated genotype lies with respect to expression levels of a focal genotype.

Congruent with our formulations of evolvability measures, as described above, the definition of global sensitivity in a dynamical system is bounded by the magnitude of parameter changes. And, similarly to our rationale for developing an evolvability measure for arbitrarily large mutations, we developed a similar statistical expression for robustness towards large mutations.

In short, this measure captures how similar the expression level of mutated genotypes remain to a focal phenotype. This is quantified by imposing a weighting function around the steady state value of the focal genotype, with perturbations around the genotype assigned probability values specified by this function. Robustness is defined as the mean value of the set of parameter perturbations around the focal genotype mapped onto a normal distribution $N_{r}(\mu_{r},\sigma_{r}^{2})$, with *μ*_*r*_ set equal to the steady state value, and a *σ*_*r*_ value that reflects the standard deviation of the parameter fluctuations used to calculate evolvability (see Figure A in S1 File). Thus, this alternative robustness measure scores phenotype neighbors by their distance to a focal phenotype under fluctuations in the genotype, such that neighbors with the same or nearly unchanged steady state expression level get a high score, and those further from a focal phenotype get a lower score, based on the mapped Gaussian. The robustness of a focal genotype with respect to a certain perturbation size is then the mean of all phenotypic neighbor scores.

One assumption that needs to be made for this alternative robustness measure to be consistent concerns the width of the score function, i.e. the value of *σ*_*r*_. If *σ*_*r*_ is small, then the robustness measure is strict and only genotypic fluctuations that lead to phenotypes that are extremely close to the focal phenotype result in high robustness. On the other hand, if *σ*_*r*_ is large, nearly all genotypes are relatively robust, since even large deviations from a focal phenotype would earn a nonzero score. Assuming that the magnitude of fluctuations *m*_*f*_ applied to a genotype correlates with the magnitude of deviation from a corresponding phenotype, we set *σ*_*r*_ = *σ*(*m*_*f*_). Thus, if the measure is based upon 10% perturbations to a genotype, we would use *σ*_*r*_ = 0.1 in the scoring normal distribution; with 20% perturbations we would use *σ*_*r*_ = 0.2.

### Evolutionary simulations

Evolutionary simulations of the auto-activation model followed the implementation described in [29]. A population of 1000 cells was considered. At the start of each simulation, the population was taken to be homogeneous with initial parameters set to the following values: *a* = 1; *k*_1_ = 0.02; *k*_2_ = 0.2; *k*_3_ = 0.1; *k*_4_ = 0.1; *k*_5_ = 0.002; *k*_*D*_ = 50 and *N* = 0. The population was modeled in a fluctuating environment that switched between selection for high and low protein levels at a constant rate *v* = 0.05 (corresponding to an environmental switch every 20 generations) for a total of 5000 generations. Fitness under the two environments (*w*_*high*_ and *w*_*low*_) was defined as

$$\begin{array}{c} \begin{array}{cc} w_{high} & =\frac{{\left(P_{end}/50\right)}^{5}}{1+{\left(P_{end}/50\right)}^{5}},\\ w_{low} & =1-w_{high}, \end{array} \end{array}$$ (16)

where *P*_*end*_ is the amount of *P* in a cell at the end of a generation, evaluated using Gillespie’s stochastic simulation algorithm run for 2000 time units. Initial conditions for the simulations were *M* = *P* = 0. New populations were produced by randomly drawing a cell from the population, and then cloning it into the new population if it had a fitness value above a random number drawn from U(0,1). This procedure was repeated until the new population consisted of 1000 cells, thereby maintaining a constant population size. Mutations were assumed to occur at a rate *μ*, which for all simulations was set to the value 0.01. Mutations were performed by adding a normally distributed random variable to the current parameter value, with the exception of mutations to the Hill coefficient *N* which were implemented by adding or subtracting 0.5 with equal probability. All parameter values were restricted to be positive. Robustness and evolvability for genotypes arising in the evolutionary simulations were computed using the same genotypic fluctuation sizes as in the G-P mapping they were compared to: robustness against small fluctuations versus evolvability based on 20% perturbations around focal genotypes. Figure G in S1 File shows the relationship between evolvability and robustness for a set of typical evolutionary simulations generated using this method.

### Simulations and software

Genotype-phenotype mappings were constructed using custom software developed in Scientific Python. Steady state values were computed using iterative approximation algorithms, as implemented in the Scientific Python module *scipy.optimize*. Evolutionary simulations were coded in the C language, and simulation code was taken from [29]. All computations were carried out on desktop computers and a Sun Grid Engine high-performance computing cluster.

## Results

To study the relationship between robustness and evolvability at the genotype level, we focus on commonly observed gene regulatory network architectures. These allow a tractable definition of both genotype and phenotype. In particular, we define the kinetic reaction rates controlling gene transcription and translation and gene/protein degradation to be the genotype, and the corresponding steady state expression level to be the phenotype. These definitions allow genotypes to be varied, while ensuring each genotype is mapped onto a specific phenotype. Furthermore, they allow us to develop mathematically rigorous measures for robustness and evolvability that are independent of organismal fitness. These are described in detail in Materials and Methods.

Based upon these measures, we evaluated the robustness and evolvability of several million genotypes sampled from two canonical gene regulatory network architectures (see Materials and Methods). Each network involves a single focal gene, whose expression is regulated either by its own gene product (circuit I) or by an upstream transcription factor (circuit II) (Fig 1). Both of these network architectures are commonly observed in nature, and are also exploited in synthetic biology as functional motifs [33, 34]. Besides their biological relevance, these network architectures also allow us to directly test the role of nonlinearity in determining the robustness-evolvability relationship. The level of nonlinearity in system dynamics resulting from these networks is dependent on kinetic rates, and as such is a property of the genotype. While circuit I permits bistability, circuit II is strictly monostable—i.e. there are no possible bistable genotypes in circuit II.

### Nonlinearity decouples robustness from evolvability

**Fig 2:**
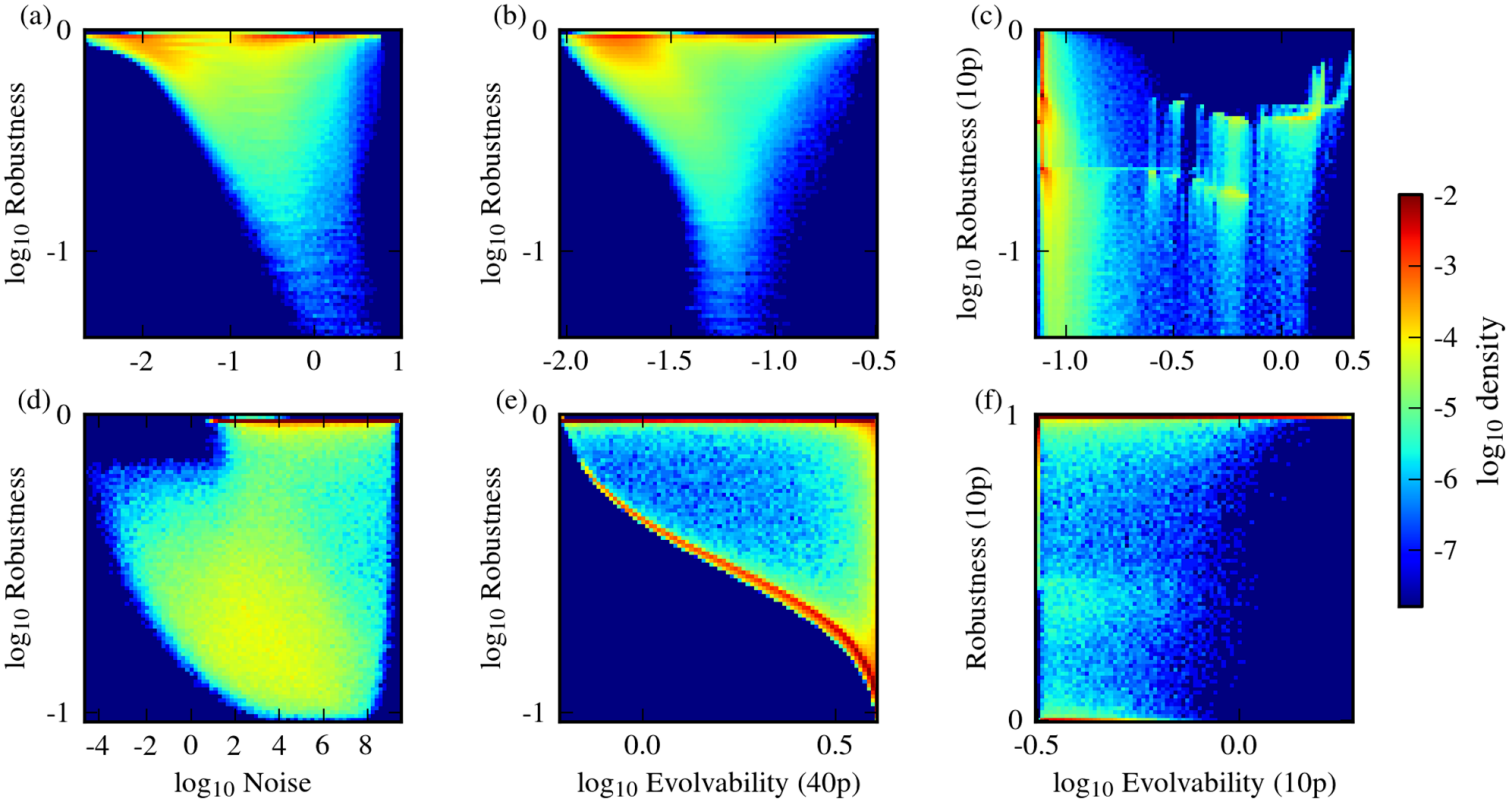
Robustness and evolvability in G-P mappings. G-P mappings of circuit I (a-c) and circuit II (d-f), showing the density of genotypes on the map. Three different combinations of robustness/evolvability measures are shown in each case. The first column (a and d) shows the relationship between sensitivity-based robustness and scaled intrinsic noise (small mutational effects); the second column (b and e) shows sensitivity-based robustness against evolvability computed using 1-mutant neighbors, represented by 40% parameter perturbations on average (small mutational effects versus large mutational effects); the third column (c and f) shows robustness against evolvability when both are computed using 10% parameter perturbations (large mutational effects). Red colors indicate areas on the G-P mapping that are highly populated by genotypes.

**Fig 3:**
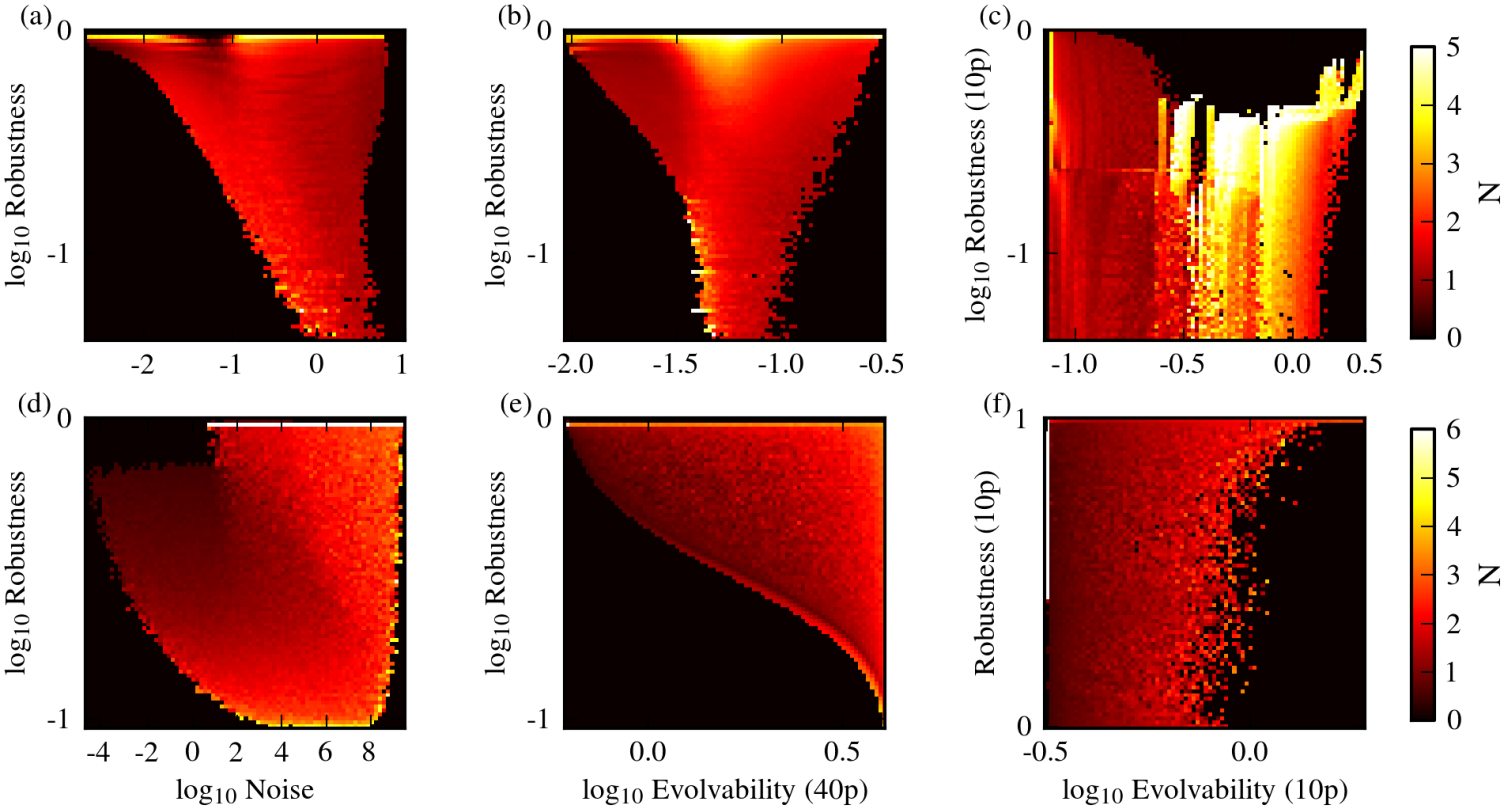
Nonlinearity in G-P mappings. Values of the nonlinearity parameter *N* on the G-P mappings of circuit I (a-c) and circuit II (d-f). The combinations of robustness/evolvability measure shown are identical to those used in Fig 2. In each plot, brighter colors signify higher mean values of *N*.

**Fig 4:**
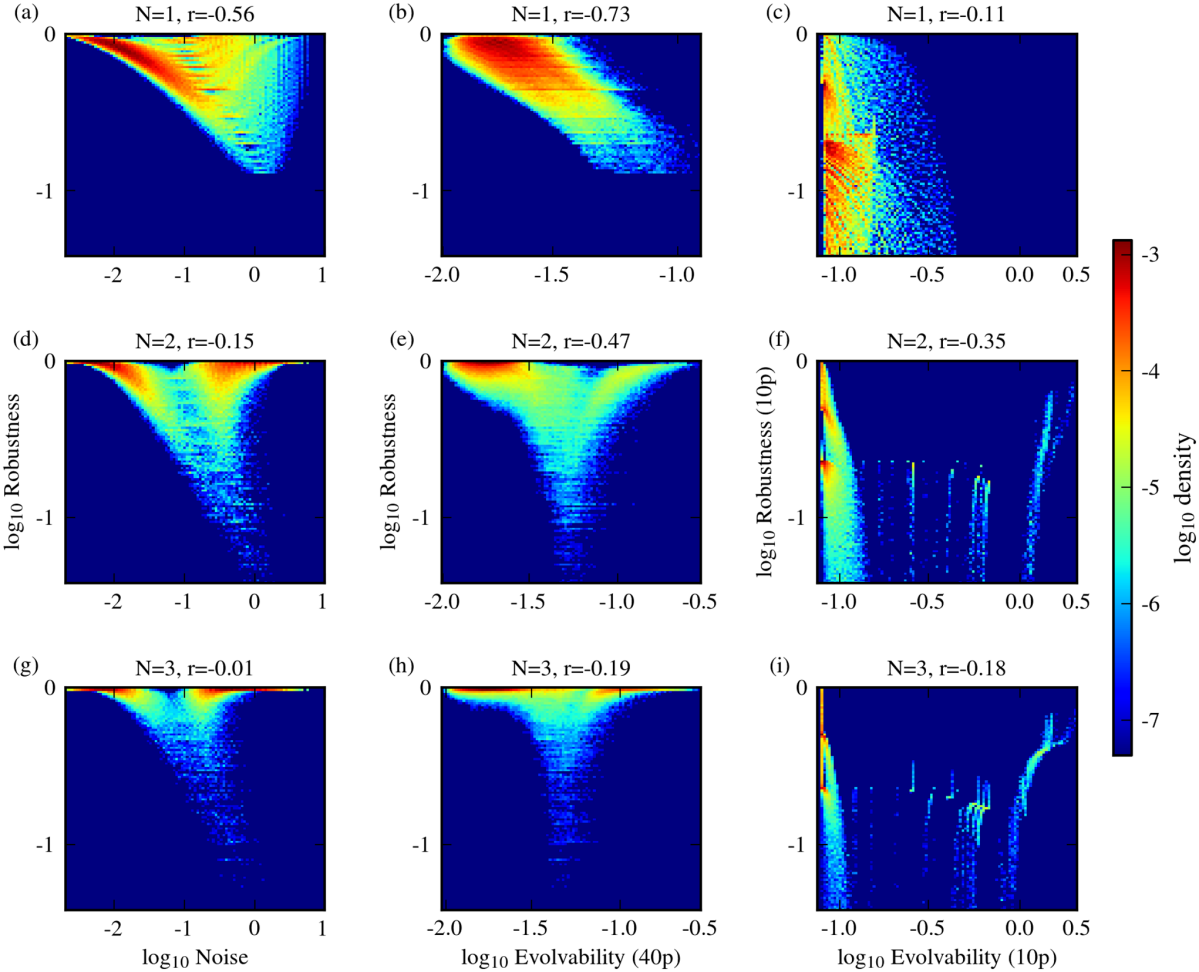
Nonlinearity shapes robustness-evolvability correlations. The G-P mapping of circuit I for different values of *N*. The first column (a,d,g) shows the relationship between scaled intrinsic noise and sensitivity-based robustness; the second column (b,e,h) shows sensitivity-based robustness versus evolvability, when evolvability is computed using large-scale parameter perturbations (1-mutant neighbors); the third column (c,f,i) shows robustness against evolvability when both are computed using 10% parameter perturbations. The Pearson correlation coefficient *r* is displayed in the panel titles. Colors indicate genotype density in each plot (cf. Figure I in S1 File for the equivalent analysis for circuit II).

For each of the two network architectures considered, we evaluated the robustness and evolvability of genotypes using combinations of the different measures defined above. For both network architectures, and for all combinations of measures used, we found that most genotypes exhibit a negative correlation between robustness and phenotypic variability (Fig 2). In all cases studied, however, there were also a substantial number of genotypes that displayed high robustness and high evolvability (Fig 2). We found that these robust and evolvable genotypes display high values of the model parameter *N*, which dictates the cooperativity of transcriptional regulation, and hence the level of nonlinearity in the system dynamics (Fig 3). Cooperativity maps the concentration of transcription regulators to the concentration of transcriptional product concentration, and for positive cooperativity (*N* > 1), this relationship between regulator and product concentration gets increasingly less gradual and more ultrasensitive with increasing *N*. When we considered genotypes that have a specific value of *N*, the effect of nonlinearity became even more apparent. For low *N*, the correlation between robustness and evolvability is moderately to strongly negative. With increasing values of *N*, however, this negative correlation reduces and eventually breaks, thereby decoupling robustness from evolvability and noise (Fig 4). This observation holds for all measurement combinations used. High *N* can lead to bistability in circuit I (Fig 1a), but not in circuit II (Fig 1b). Thus, bistability alone cannot explain the observed decoupling of robustness and phenotypic variability as we have only considered monostable genotypes in circuit I.

### The decoupling effects of nonlinearity arise from the generation of regions presenting sudden changes in phenotype with small changes in genotype

**Fig 5:**
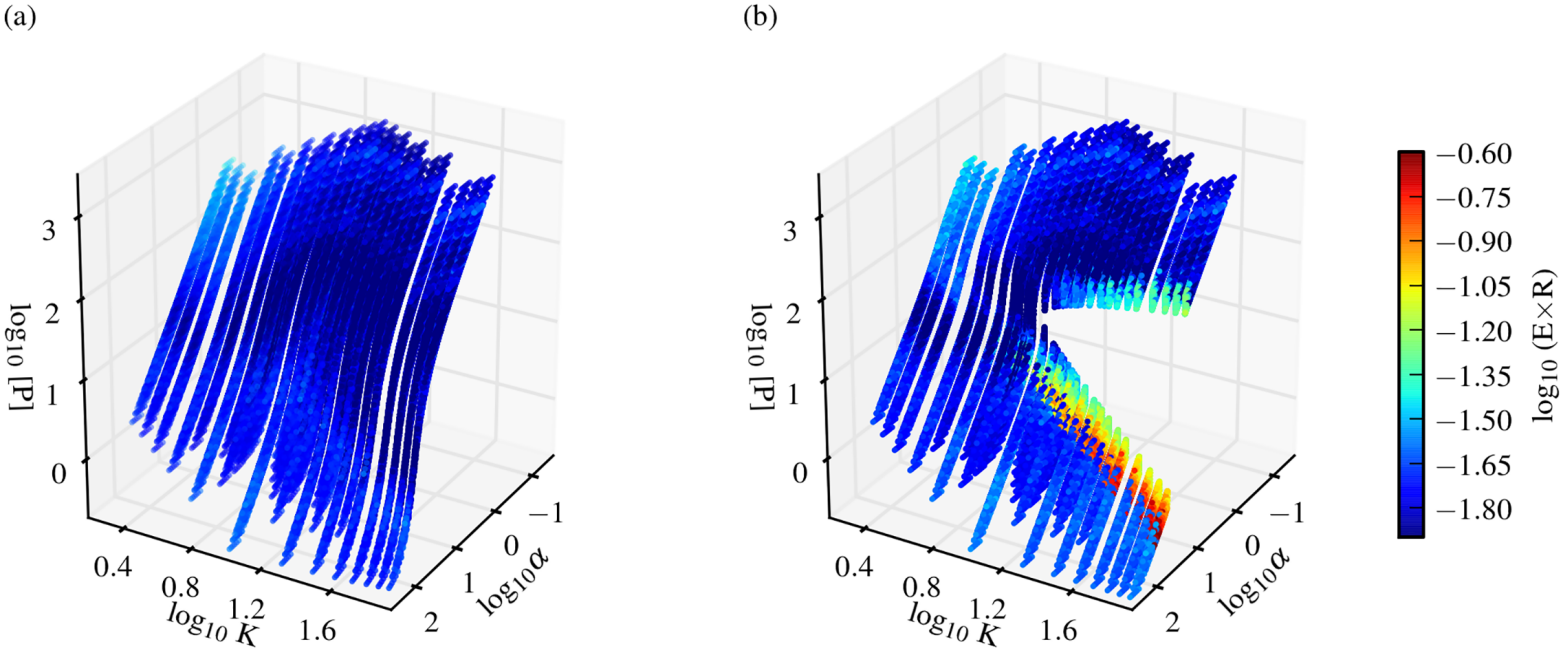
Nonlinearity creates regions presenting sudden changes in phenotype with small changes in genotype. Expression level as a function of the composite parameters *α* and *K* for the G-P mappings of circuit I obtained with *N* = 1 (panel a) and *N* = 2 (panel b). The product, (*E* × *R*), of evolvability computed for large scale perturbations (1-mutant neighbors) and sensitivity-based robustness is color-coded, with blue indicating a negative E/R correlation and red indicating the region of high robustness and evolvability (cf. Figure B in S1 File).

To achieve a more complete mechanistic understanding of the effect of different parameters on the robustness and evolvability relationship, for both circuits we derived composite parameters that simplified the analysis of robustness (see Eqs (5) and (9). For circuit I, the composite parameter *α* combines production and decay rates of all components, and is inversely related to the steady state expression level, such that high *α* values correspond to low steady state expression levels. For circuit II, an analogous composite parameter *β* can be derived. For both networks, a second composite parameter *K* captures the ratio of regulation-mediated transcription to basal transcription, *k*_2_/*k*_1_. As with the nonlinearity parameter *N*, we find that these composite parameters show specific distributions for genotypes that break the negative correlation between robustness and evolvability (Figures C, D in S1 File). In particular, robust and evolvable genotypes tend to display high *K* and *α* (*β*) values. When we plotted steady state expression levels (i.e. phenotypes) for genotypes with different values of *N* against *α* and *K* for circuit I, we found that the parameter combinations yielding robust and evolvable genotypes map onto the edges of regions presenting sudden changes in phenotype with small changes in genotype (Fig 5). These regions correspond to regions of drastic change in expression levels and could be described as ‘phenotypic cliffs’. The composite parameters, as well as *N*, contribute to the generation of these regions. The genotypes with parameter sets characterized by high *K* and *α* (*β*) values are located at the base of these regions, and it is these genotypes that can achieve high robustness and evolvability. They can maintain high robustness with regard to some mutations (i.e. those pushing them away from these regions), but achieve high phenotypic diversity with regard to others (i.e. those pushing them beyond these regions’ boundaries), even when these mutations are of the same magnitude (Figures C–E in S1 File). The color coding in Fig 5 shows the product of robustness and evolvability (*E* × *R*). This product gets larger as both robustness and evolvability are increased, hence genotypes with high values of (*E* × *R*) are shown to be at the base of these regions.

### Robust and evolvable genotypes occur in regions of parameter space where system parameters can be freely tuned without affecting robustness

**Fig 6:**
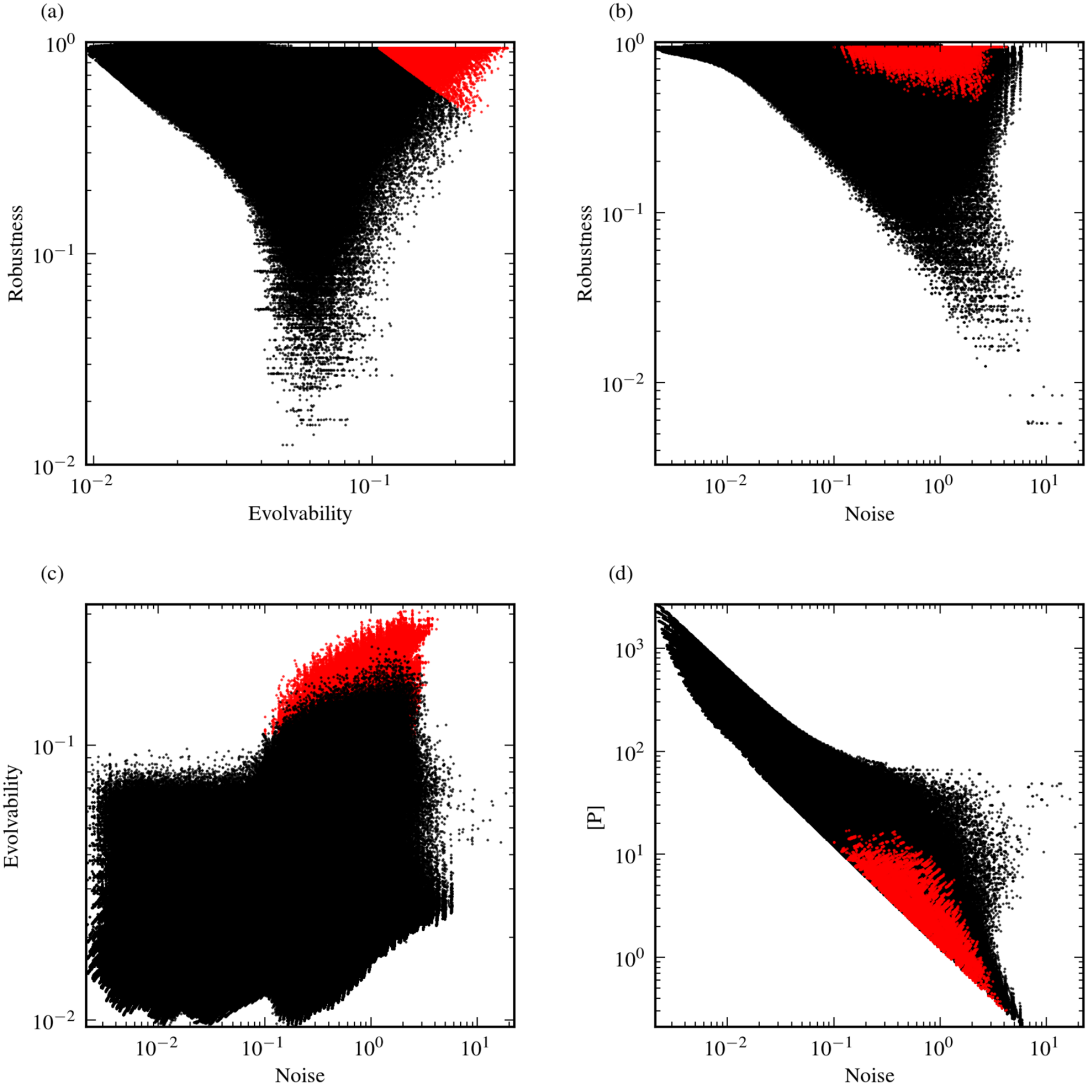
Robustness, evolvability, noise and expression levels for genotypes that are robust and evolvable. The relationships between sensitivity-based robustness, evolvability computed using 1-mutant neighbors, noise and gene expression levels in circuit I, when only monostable genotypes are considered. Genotypes from the set of genotypes characterized by high robustness and evolvability ((*E* × *R*) > 0.1, cf. Figure B in S1 File) are drawn in red to compare their positions with respect to different measure combinations. (a): The robustness-evolvability relationship of the G-P mapping. (b): Scaled intrinsic noise versus robustness. (c): The relationship between scaled intrinsic noise and evolvability. (d): Gene expression levels at steady state ($\bar{P}$) plotted against scaled intrinsic noise.

As described above, genotypes found near phenotypic cliffs are thus characterized by high *K* and *α* (*β*) values. The high values of *α* (*β*) result in low expression levels, explaining the observation that genotypes with high levels of both robustness and evolvability on Fig 2 (cf. also Fig 4) are also those with low expression (Figure E in S1 File). This is an unexpected result, especially when we consider mutations with infinitesimally small effects. For such mutations, the relationship between robustness and phenotypic variance (i.e. noise) corresponds to a characteristic of stochastic gene expression: mutations that decrease the mean expression level are expected to increase noise and vice versa. We find that this intuition holds for genotypes with low *N* values, with robustness and noise exhibiting a clear negative correlation in this case (Fig 4a). The trend is, however, clearly broken by genotypes displaying nonlinear system dynamics and ultrasensitivity, given by high *N* and *K* values (Fig 4d and 4g). To better understand the effects of these parameters, we derived an analytical expression for the sensitivity-based robustness measure (see the Supplementary Information (S1 File)). This shows that the robustness measure becomes largely independent of *K* and *α* (*β*) as *N* is increased. Hence in the parameter regimes yielding ultrasensitivity in gene expression levels as response to levels of transcription factors, the system parameters can be freely tuned without affecting the robustness levels (see S1 File). This mechanism could allow for the observed robust, yet noisy genotypes seen in Fig 4. This same mechanism could also explain the results from mutations with arbitrarily large effects, as the measures for quantifying evolvability under small and large mutational effects (i.e. expression noise and expression variability) display a significant correlation (Fig 6).

### Robust and evolvable genotypes also emerge from *in silico* evolution under fluctuating selection

**Fig 7:**
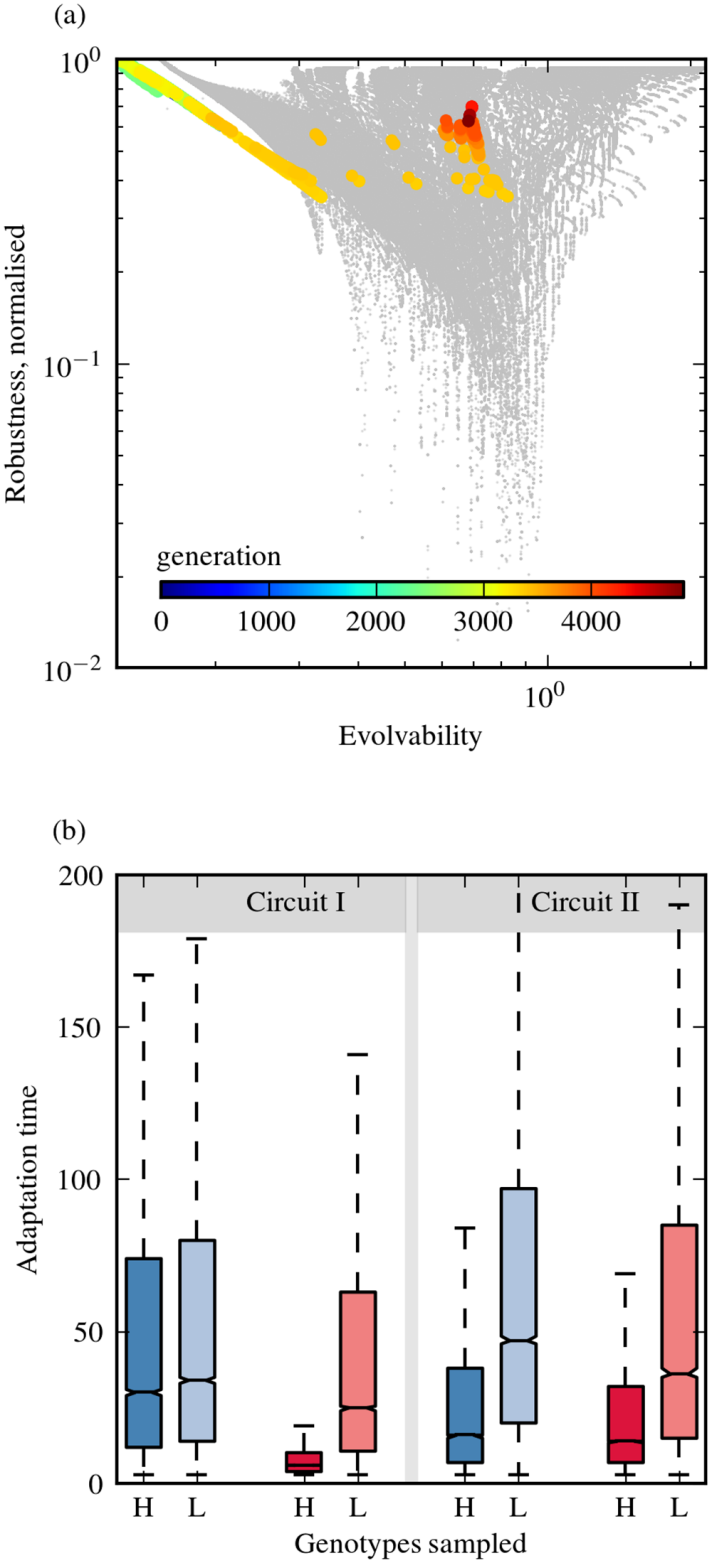
*In silico* simulation under fluctuating environments and adaptation time for genotypes that are robust and evolvable. (a): Monostable genotypes corresponding to the population mean of parameter values arising from different generations of a single *in silico* simulation under fluctuating environments, superimposed onto the G-P mapping of circuit I (grey backdrop). Genotypes derived from each generation are color-coded according to generation number, with red indicating the oldest generations. For both the G-P mapping and the *in silico* simulations, evolvability was computed using parameter perturbations of ±20% (cf. Figure F in S1 File)), and robustness was calculated using the sensitivity-based measure. See also Figure G in S1 File, which shows the results obtained for other runs of the evolutionary simulation algorithm. (b): Box plots showing adaptation time for genotypes exhibiting high evolvability (*H*) and low evolvability (*L*) under two selection scenarios: selection for a 10% reduction (blue) and for a 10% increase (red) in steady state expression levels. For circuit I, high evolvability (*H*) was *E* > 0.7 and low evolvability (*L*) was *E* < 0.1 on G-P mappings using robustness and evolvability measures computed from 10% parameter perturbations (cf. Fig 2c). For circuit II, genotypes from *H* had *E* > 1.2 and genotypes from *L* had *E* < 0.6 based upon the same measures (cf. Fig 2f). For both circuits, 11000 genotypes were picked from each respective region in the G-P mapping. For both circuits, adaptation times for the two regions differed significantly (*p* < 0.01, two-sample Kolmogorov-Smirnov test, n = 11000).

Given that we adopted fitness-independent measures of evolvability, and evaluated these for individual genotypes, it is not clear if the robust and evolvable genotypes identified here would display increased evolutionary performance under population dynamics and different fitness functions. In order to understand this, we performed two analyses. Firstly, we ran *in silico* evolutionary simulations using a specific function relating expression level to fitness and under fluctuating selection (see Methods): one environment selecting for high expression levels, and one selecting for low expression. The two environments switched every 20 generations. It was previously shown that environmental fluctuations at this rate, combined with an appropriate mutation rate, promote the speed of adaptation [29]. Using the same settings as in that study, we ran here evolutionary simulations for 5000 generations using a population size of 1000 (see Methods). For the resulting evolved genotypes, we evaluated their evolvability and robustness using our fitness-independent measures (Fig 7a and Figure G in S1 File). We found that the resulting evolved genotypes all have high values of *N*, *α* and *K*, indicating that they occupy a similar region of the genotype space as the robust and evolvable genotypes identified from this analysis (Fig 5). This shows that evolutionary simulations, run under conditions selecting for evolvability [29], result in genotypes that would be identified as evolvable and robust based on the fitness-independent measures presented here. Secondly, we evaluated the speed of evolution of new phenotypes from a given genotype by measuring the number of mutations required to achieve a 10% increase/decrease in expression level. To do this, we assumed a low mutation rate—large population size limit, where the evolutionary dynamics can be approximated by a random walk dominated by the fixation of a fitter mutant [42]. We implemented such random walks by starting from a given genotype, evaluating single mutations created by randomly perturbing a chosen parameter, and then accepting mutants with a certain improvement in expression level as the new wild type (see Materials and Methods). Performing several such random walks, starting from genotypes chosen from regions of high or low levels of fitness-independent evolvability, we counted the number of mutations needed to achieve a 10% increase/decrease in expression level. The genotypes chosen were mutated with the same perturbation size that was used to compute robustness and evolvability as shown in Fig 2c and 2f, making all measures consistent with each other. We found that robust and evolvable genotypes displayed shorter adaptation times for both selection scenarios in both circuits (Fig 7b).

## Discussion

We have performed an extensive analysis of genotype robustness and evolvability using mathematical models of two common gene regulatory networks involving a single gene under auto- (or transcription factor) mediated regulation. We have used the expression levels of this gene as a phenotype and the system parameters controlling expression level as the genotype. Defining several complementary measures for genotype robustness and evolvability under mutations of different size, we have evaluated these properties for several million genotypes for each network architecture. This analysis revealed that for most genotypes, robustness and evolvability display a negative correlation, but there exist a significant number of genotypes for which this trade-off can be broken. This observation holds for all the combinations of the different measures utilized. Furthermore, the identified robust and evolvable genotypes using these fitness-independent measures are also found to emerge under *in silico* evolution when selection schemes that are shown to facilitate adaptation time are used. This suggests that our fitness-independent measures applied to a genotype-phenotype map are then able to identify genotypes that are evolvable in a population dynamics context and using fitness functions based on that same phenotype (such as adaptation time, or performing well in fluctuating environments, section 4 of the Results). Thus we conclude that the fitness-dependent and the fitness-independent view on evolvability need not be mutually exclusive.

We show that the robust and evolvable genotypes found in this analysis are characterized by parameter combinations that confer nonlinearity and ultrasensitivity in system dynamics. Among the system parameters that can determine whether a given genotype confers these characteristics, we find the strongest effect to come from the parameter controlling nonlinearity. This effect extends to the point that the breaking of the robustness and evolvability trade-off is only observed when a certain threshold level of nonlinearity is exceeded. We find that this strong effect comes from the fact that nonlinear system dynamics allow for the emergence of phenotypic cliffs in the genotype-phenotype map, and thereby enable the presence of genotypes that can be robust and evolvable. This result is corroborated by experimental studies on transcriptional circuits controlled by LexA in *E. coli*, which show that nonlinearity stemming from negative feedback brings about the ability to withstand mutational effects (i.e. robustness), while at the same time enabling capacitance of the system (i.e. evolvability)[43]. This finding is dependent on nonlinear dynamics: upon removal of negative feedback, both mutational robustness and evolvability are reduced. Here, we show the mechanistic basis of the relationship between these two features by directly tuning the degree of nonlinearity in a given architecture.

It is evident that the results presented here need to be considered in the context of the robustness and evolvability measures we applied. These measures were chosen to capture the properties of individual genotypes (rather than populations) under different mutational effects, and without invoking reference to organismal fitness. The relationships we found between the different measures of robustness and evolvability are in line with experimental findings. In particular, the correlation we observe between the evolvability measure defined for mutations of large effect and intrinsic noise fits with the empirical finding of a high correlation between environmental plasticity and gene expression noise in yeast [25, 32].

Gene regulation circuits can and frequently do exhibit more complex architectures than the two architectures covered in our study. The latter have, however, gained much attention previously, for instance as model systems for implementation in synthetic biology [44, 45]. Restricting our analysis to these well-researched architectures has the valuable benefit of tractability concerning their mechanistic underpinnings. By focusing on such minimal architectures, we are able to restrict the search space for potential mechanisms which can affect the relationship between robustness and evolvability. Thus, we find that even the simplest cases of nonlinear gene expression circuits are capable of solving the robustness-evolvability trade-off.

Our findings provide a genotype-based resolution of the robustness-evolvability trade-off, and do not contradict previous suggestions based on population dynamics [6, 8, 12–16, 18].

In particular, our results relate to a recent population genetics study suggesting that there can be an optimal level of robustness that promotes evolvability, depending on the properties of the fitness landscape [15]. Using fitness landscapes with specified statistical properties, that study found that while robustness is negatively correlated with evolvability when mutations allow access to any of the possible phenotypes, there can be a positive correlation between robustness and evolvability when mutations can allow access to only a small fraction of all phenotypes. In light of those findings, it is interesting to see that our biologically well-defined genotype-phenotype mapping contains robust and evolvable genotypes only at increasing levels of nonlinearity in the equations governing gene expression levels. It is possible that increasing nonlinearity re-shapes this genotype-phenotype mapping in a way that is in line with the statical assumptions made in [15]. Furthermore, we find that robust and evolvable genotypes identified in our analysis occupy specific regions of the genotype-phenotype map, characterized by sudden changes in phenotype with small changes in genotype. These genotypes also present a specific level of nonlinearity and other kinetic parameters. This could relate to them being “tuned” to have a specific level of robustness as suggested in [15]. This proposition is also reminiscent of the idea of biological systems being in a critical state that enhances their evolvability [21–23, 46], but does not rely on the presence of chaos in system dynamics.

It is also possible to draw an analogy between the findings presented here and changes observed when considering a system passing through distinct dynamical regimes due to parameter alterations [47]. It must be noted, however, that in our study the analysis is restricted to a single dynamical regime. In particular, the finding that the ability of nonlinearity to break the robustness-evolvability trade-off extends to systems that do not allow for bistability shows that proximity to a bifurcation surface is not a necessary condition for genotypes to be robust and evolvable at the same time.

The mechanistic insights gained from this study could provide an improved explanation for the emergence of evolvability in laboratory evolution experiments [48]. In particular, our findings would suggest that genetic mutations identified in these studies could relate to their effects on noise and nonlinearity in gene regulation. Future experimental studies towards confirming this suggestion will significantly improve our understanding of how natural gene regulatory systems achieve robustness and evolvability, and allow us to better design robust synthetic gene regulatory circuits [49].

## Conclusion

The question of how biological systems are able to withstand mutational changes (robustness), yet still remain able to produce phenotypic variation (evolvability) has gained considerable interest in recent years. Despite important insights towards the understanding of scenarios that allow biological networks to be both robust and evolvable, the mechanistic underpinnings of such phenomena are still elusive. By performing mathematical analyses of common gene regulatory network motifs, we provide a mechanistic explanation of the robustness-evolvability trade-off. Using several measures across scales of perturbation, we find that nonlinearity consistently breaks the predominant negative correlation found between robustness and phenotypic variability. This holds for both small and large perturbations in genotypes with low expression levels. Our results provide a potential link between the abundance of nonlinearity in biological regulatory networks and their apparent ability to be both robust and evolvable at the same time.

## Supporting Information

S1 File Supplementary information containing detailed mathematical analyses of gene regulation networks, as well as supplemental figures and tables. (PDF)
