# Supplementary material for: Nonlinear Dynamics in Gene Regulation Promote Robustness and Evolvability of Gene Expression Levels
Source: PLoS One. 2016 Apr 15;11(4):e0153295. doi: 10.1371/journal.pone.0153295 (PMC4833316; doi:10.1371/journal.pone.0153295)
Supplement: S1 File — (PDF) [file pone.0153295.s001.pdf]

Supplementary Information for *Nonlinear dynamics in gene regulation promote robustness and evolvability of gene expression levels.*, A. Steinacher *et al.*

Contents

S1 Analysis of the auto-activation model (circuit I) 1
S1.1 Steady states 2
S1.2 Stabilities of the steady states 2
S1.3 Sensitivity-based robustness 3

S2 Analysis of the simple-activation model (circuit II) 6
S2.1 Steady states 6
S2.2 Stabilities of the steady states 6
S2.3 Sensitivity-based robustness 7

References 8

S3 Supplementary Tables 9

S4 Supplementary Figures 11

S1 Analysis of the auto-activation model (circuit I)

Recall from the main paper that the ordinary differential equations describing the dynamics of mRNA (M) and protein (P) in the auto-activation circuit are

$$\begin{aligned} \frac{d[M]}{dt} &= F_1([M],[P]) = \frac{a\left(k_1 + k_2\left(\frac{[P]}{k_D}\right)^N\right)}{1+\left(\frac{[P]}{k_D}\right)^N} - k_4[M], \\ \frac{d[P]}{dt} &= F_2([M],[P]) = k_3[M] - k_5[P], \end{aligned} \tag{S1}$$

where the parameters a, k_1 and k_2 determine the transcription rate, k_3 denotes the translation rate, k_4 and k_5 are degradation rates, N specifies the degree of cooperativity in the feedback loop and k_D is the half-activation parameter. By letting $\mathbf{x} = ([M],[P])^T$ and $\mathbf{F}(\mathbf{x}) = (F_1(\mathbf{x}), F_2(\mathbf{x}))^T$ denote the state vector and vector field respectively of (S1), this can be written in the vectorised form

$$\frac{d\mathbf{x}}{dt} = \mathbf{F}(\mathbf{x}).$$

S1.1 Steady states

It follows from (S1) that the steady state protein level $\overline{P}$ is the solution of the equation

$$\frac{a\left(k_1 + k_2\left(\frac{\overline{P}}{k_D}\right)^N\right)}{1 + \left(\frac{\overline{P}}{k_D}\right)^N} = \frac{k_4 k_5}{k_3}\overline{P}, \tag{S2}$$

while the corresponding steady state mRNA level $\overline{M}$ is given by

$$\overline{M} = \frac{k_5}{k_3}\overline{P}.$$

To simplify the subsequent analysis, we introduce the nondimensionalised protein variable

$$X = \frac{\overline{P}}{k_D}, \tag{S3}$$

together with the composite parameters α and K defined below:

$$\alpha = \frac{k_D k_4 k_5}{a k_1 k_3}, \quad K = \frac{k_2}{k_1}. \tag{S4}$$

It then follows from (S2) that the equation for steady state protein level in terms of X is

$$f\left(X\right) = \alpha X, \tag{S5}$$

where the function $f : \mathbb{R} \to \mathbb{R}$ is defined as

$$f\left(x\right) = \frac{1 + Kx^N}{1 + x^N}. \tag{S6}$$

For $K > 1$, the function $f\left(x\right)$ is monotonically increasing and saturates at K for large x.[1] As N increases through 1, $f\left(x\right)$ switches from a curve with hyperbolic shape to a sigmoid. By considering how the graphs of $f\left(x\right)$ and αx vary with α for $x \geq 0$, it is clear that for $\alpha \gg 1$ and $\alpha \ll 1$ there is only a single steady state, while for intermediate values of α, there will generically be one or three steady states depending on the exact values of K and N.

S1.2 Stabilities of the steady states

The stability of a steady state $\left(\overline{M}, \overline{P}\right)$ is determined by the eigenvalues of the corresponding Jacobian matrix of partial derivatives $\frac{\partial \mathbf{F}}{\partial \mathbf{x}}(\overline{M}, \overline{P})$, with $\left(\overline{M}, \overline{P}\right)$ stable if both eigenvalues have negative real part [2]. The Jacobian is calculated from (S1) as

$$\frac{\partial \mathbf{F}}{\partial \mathbf{x}}\left([M], [P]\right) = \begin{pmatrix} -k_4 & \frac{aN(k_2 - k_1)\left(\frac{[P]}{k_D}\right)^N}{[P]\left(\left(\frac{[P]}{k_D}\right)^N + 1\right)^2} \\ k_3 & -k_5 \end{pmatrix}.$$

[1]Note: as we are considering an auto-activation circuit, $K > 1$ by assumption.

At a steady state $\left(\overline{M}, \overline{P}\right)$, the Jacobian can be written in terms of X as

$$\frac{\partial \mathbf{F}}{\partial \mathbf{x}}\left(\overline{M}, \overline{P}\right) = \begin{pmatrix} -k_4 & \frac{ak_1}{k_D} f'(X) \\ & \\ k_3 & -k_5 \end{pmatrix}, \tag{S7}$$

where

$$f'(x) = \frac{N(K-1)x^{N-1}}{(1+x^N)^2}. \tag{S8}$$

The eigenvalues of the Jacobian satisfy

$$\lambda^2 + (k_4 + k_5)\lambda + k_4 k_5 \left(1 - \frac{f'(X)}{\alpha}\right) = 0.$$

Solving this quadratic equation yields

$$\lambda_\pm = \frac{-(k_4 + k_5) \pm \sqrt{(k_4 + k_5)^2 - 4k_4 k_5 \left(1 - \frac{f'(X)}{\alpha}\right)}}{2}. \tag{S9}$$

Eqn. (S9) shows that the stability of $\left(\overline{M}, \overline{P}\right)$ depends on the sign of $f'(X) - \alpha$. We can therefore distinguish three possibilities:

1. $f'(X) < \alpha$. In this case, $\lambda_- < \lambda_+ < 0$, and so $\left(\overline{M}, \overline{P}\right)$ is stable.
2. $f'(X) > \alpha$. In this case, $\lambda_- < 0 < \lambda_+$, and so $\left(\overline{M}, \overline{P}\right)$ is unstable.
3. $f'(X) = \alpha$. This case corresponds to a saddle-node bifurcation ($\lambda_+ = 0$, $\lambda_- = -\left(k_4 + k_5\right)$) at which two steady states are created, one stable and the other unstable [2]. The bifurcation occurs when the graph of the line αx is tangent to the graph of $f(x)$. It is straightforward to show that equations (S6) and (S8) together imply that $N > 1$ and $K > 1$ are necessary conditions for the saddle-node bifurcation to occur.

It thus follows from considering intersections of the graph of $f(x)$ with the line αx that for parameter values yielding a single steady state $\left(\overline{M}_0, \overline{P}_0\right)$, this steady state is always stable (monostability). For parameter values yielding three steady states $\left\{\left(\overline{M}_i, \overline{P}_i\right) : i = 1, 2, 3\right\}$ with $\overline{P}_1 < \overline{P}_2 < \overline{P}_3$, $\left(\overline{M}_2, \overline{P}_2\right)$ is unstable while $\left(\overline{M}_1, \overline{P}_1\right)$ and $\left(\overline{M}_3, \overline{P}_3\right)$ are both stable (bistability).

S1.3 Sensitivity-based robustness

Recall from the main paper that for small parameter perturbations, robustness is defined as inverse sensitivity $1/S$, where S measures the effect of the perturbations on steady state protein levels:

$$S = \left\| \frac{\partial \ln \overline{P}}{\partial \ln \mathbf{k}} \right\|_2^2. \tag{S10}$$

The derivative $\frac{\partial \ln \overline{P}}{\partial \ln \mathbf{k}}$ in (S10) is given by

$$\frac{\partial \ln \overline{P}}{\partial \ln \mathbf{k}} = \begin{pmatrix} 0 & 1 \end{pmatrix} \begin{pmatrix} \frac{1}{\overline{M}} & 0 \\ 0 & \frac{1}{\overline{P}} \end{pmatrix} \frac{\partial \overline{\mathbf{x}}}{\partial \mathbf{k}} \begin{pmatrix} a & 0 & \cdots & 0 \\ 0 & k_1 & \ddots & \vdots \\ \vdots & \ddots & \ddots & 0 \\ 0 & \cdots & 0 & N \end{pmatrix}, \tag{S11}$$

where $\overline{\mathbf{x}} = \left(\overline{M}, \overline{P}\right)^T$, and the vector $\mathbf{k} = (a, k_1, k_2, k_3, k_4, k_5, k_D, N)^T$ gathers the model parameters. $\frac{\partial \overline{\mathbf{x}}}{\partial \mathbf{k}}$ can be computed by considering the steady state equation

$$\mathbf{F}\left(\overline{\mathbf{x}}\right) = \mathbf{0}.$$

Differentiating both sides of the equation with respect to $\mathbf{k}$ and rearranging yields

$$\frac{\partial \overline{\mathbf{x}}}{\partial \mathbf{k}} = -\left(\frac{\partial \mathbf{F}}{\partial \mathbf{x}}\left(\overline{\mathbf{x}}\right)\right)^{-1} \frac{\partial \mathbf{F}}{\partial \mathbf{k}}\left(\overline{\mathbf{x}}\right). \tag{S12}$$

$\frac{\partial \mathbf{F}}{\partial \mathbf{x}}\left(\overline{\mathbf{x}}\right)$ was previously computed in eqn. (S7), while for a given point $\mathbf{x} = ([M], [P])^T$, the matrix of partial derivatives of the vector field w.r.t. parameters, $\frac{\partial \mathbf{F}}{\partial \mathbf{k}}\left(\mathbf{x}\right)$, has the form

$$\frac{\partial \mathbf{F}}{\partial \mathbf{k}}\left([M], [P]\right) = \begin{pmatrix} \frac{k_1 + k_2\left(\frac{[P]}{k_D}\right)^N}{1+\left(\frac{[P]}{k_D}\right)^N} & 0 \\ \frac{a}{1+\left(\frac{[P]}{k_D}\right)^N} & 0 \\ \frac{a\left(\frac{[P]}{k_D}\right)^N}{1+\left(\frac{[P]}{k_D}\right)^N} & 0 \\ 0 & [M] \\ -[M] & 0 \\ 0 & -[P] \\ \frac{aN(k_1-k_2)\left(\frac{[P]}{k_D}\right)^N}{k_D\left(1+\left(\frac{[P]}{k_D}\right)^N\right)^2} & 0 \\ -\frac{\ln\left(\frac{[P]}{k_D}\right)a(k_1-k_2)\left(\frac{[P]}{k_D}\right)^N}{\left(1+\left(\frac{[P]}{k_D}\right)^N\right)^2} & 0 \end{pmatrix}^T.$$

At a steady state, this matrix can be written in terms of the nondimensionalised steady state protein level X as

$$\frac{\partial \mathbf{F}}{\partial \mathbf{k}}\left(\overline{M}, \overline{P}\right) = \begin{pmatrix} \alpha k_1 X & 0 \\ \frac{a}{1+X^N} & 0 \\ \frac{aX^N}{1+X^N} & 0 \\ 0 & \frac{k_5 k_D}{k_3}X \\ -\frac{k_5 k_D}{k_3}X & 0 \\ 0 & -k_D X \\ -\frac{ak_1}{k_D}Xf'(X) & 0 \\ \frac{ak_1}{N}X\ln(X)f'(X) & 0 \end{pmatrix}^T. \tag{S13}$$

Combining (S7), (S11), (S12) and (S13) yields the required expression for $\frac{\partial \ln \overline{P}}{\partial \ln \mathbf{k}}$:

$$\frac{\partial \ln \overline{P}}{\partial \ln \mathbf{k}} = \frac{1}{\alpha - f'(X)} \begin{pmatrix} \alpha & \frac{1}{X(1+X^N)} & \frac{KX^N}{X(1+X^N)} & \alpha & -\alpha & -\alpha & -f'(X) & \ln(X) f'(X) \end{pmatrix}.$$

This gives the final expression below for the sensitivity:

$$S = \frac{1}{(\alpha - f'(X))^2} \left(4\alpha^2 + \frac{\left(KX^N\right)^2 + 1}{X^2 \left(1 + X^N\right)^2} + f'(X)^2 \left(\ln(X)^2 + 1\right) \right). \tag{S14}$$

Note that as X depends on α, K and N through equations (S5) and (S6), the sensitivity (and hence robustness) is also a function of these parameters only. Moreover for large N, it is clear from considering intersections of the graph of $f(x)$ with the line αx that either: (i) $X \approx \frac{1}{\alpha}$ (α sufficiently small); or (ii) $X \approx \frac{K}{\alpha}$ (α sufficiently large). In case (i), it follows from eqns. (S5), (S6) and (S8) that $f'(X) \approx \frac{N(K-1)}{\alpha^{N-1}}$ and $\frac{\left(KX^N\right)^2+1}{\left(1+X^N\right)^2} \approx 1$. In case (ii), it can be deduced that $f'(X) \approx \frac{N(K-1)\alpha^{N+1}}{K^{N+1}}$ and $\frac{\left(KX^N\right)^2+1}{\left(1+X^N\right)^2} \approx K^2$. Thus, in both cases, $\alpha \gg f'(X)$ and $\frac{\left(KX^N\right)^2+1}{X^2\left(1+X^N\right)^2} \approx \alpha^2$, implying that $S \approx 5$. Hence, high N levels lead to robustness having a weak dependence on α and K (and therefore also on the steady state protein level $\overline{P}$). For such genotypes, the parameters comprising α and K can be independently tuned so as to promote evolvability, whilst maintaining robustness. Increasing N therefore gives rise to the emergence of areas of the G-P mapping that contain genotypes which are both evolvable and robust (see Figure 2 of the main paper).

Table A shows the parameter distributions in the different regions of the G-P mapping constructed using the nominal parameter set from [1]. Table B shows the distributions for the G-P mapping constructed using a realistic parameter set obtained experimentally from the *E. coli lac* operon. It can be seen that for both mappings, genotypes with high evolvability and robustness exhibit increased values of α in addition to the high levels of N predicted by the analysis above. Recall from section S1.1 that α is a composite parameter containing production and degradation rates: a, k_3, k_4, k_5 and k_D (see eqn. (S4)). For α to be high, either its denominator (ak_1k_3) needs to be low, or its numerator ($k_Dk_4k_5$) needs to be high (or both), corresponding to low protein expression and high protein degradation respectively. The simplest way this can be achieved is by one of the parameters in the denominator or numerator being low or high accordingly. However, our results do not indicate any such clear tendency among single parameters within the set of highly evolvable genotypes. Indeed, we do not find individual parameters to vary as consistently as the composite parameter α in different regions of the G-P mapping. This suggests that in the regions of high robustness and evolvability, the exact values of the individual parameters comprising α are less important than their ratios. Furthermore, it follows from the analysis above that the high α values characteristic of these regions imply that $X \approx \frac{K}{\alpha}$, and so $\overline{P} \approx \frac{Kk_D}{\alpha}$ (see eqn. (S3)). These genotypes should therefore have low expression levels, and this is indeed consistent with our observations (see Figure H). Finally, the composite parameter K is found to be high for highly evolvable genotypes in the nominal G-P mapping, whereas it is slightly diminished for such genotypes in the G-P mapping based on sampling around *E.coli* parameter values. In addition, highly evolvable genotypes in the G-P mapping using parameter regimes sampled from *E. coli* are associated with high values of the half-activation parameter k_D, while k_D is relatively unchanged in the nominal parameter set mapping (see Figures J and K)

S2 Analysis of the simple-activation model (circuit II)

The model equations are, as given in the main paper,

$$\frac{d[M]}{dt} = F_1([M],[P]) = a\left(\frac{k_1 + k_2\left(\frac{T}{k_D}\right)^N}{1+\left(\frac{T}{k_D}\right)^N}\right) - k_4[M],$$
$$\frac{d[P]}{dt} = F_2([M],[P]) = k_3[M] - k_5[P], \tag{S15}$$

where mRNA and protein are represented by M and P respectively, T denotes the concentration of the transcription factor (TF), and all other parameters represent the same processes that they did previously for the auto-activation circuit in (S1).

S2.1 Steady states

At steady state, the protein level is determined by

$$\overline{P} = \frac{ak_1k_3}{k_4k_5}\left(\frac{1+\frac{k_2}{k_1}\left(\frac{T}{k_D}\right)^N}{1+\left(\frac{T}{k_D}\right)^N}\right), \tag{S16}$$

while the corresponding steady state mRNA level $\overline{M}$ is

$$\overline{M} = \frac{k_5}{k_3}\overline{P}.$$

There is therefore always only one steady state of the system $\left(\overline{M},\overline{P}\right)$. To simplify the subsequent analysis of the model, we introduce the following composite parameters (cf. (S4)):

$$\beta = \frac{k_4k_5}{ak_1k_3}, \quad K = \frac{k_2}{k_1}, \quad \gamma = \frac{T}{k_D}. \tag{S17}$$

Note that γ represents the nondimensionalised TF concentration. Also, as in the analysis of the auto-activation circuit, K is the ratio of the TF-mediated and basal transcription rates. The steady state protein level in this case can thus be written in the form

$$\overline{P} = \frac{1}{\beta}f(\gamma), \tag{S18}$$

where $f(x)$ is the function defined previously in eqn. (S6).

S2.2 Stabilities of the steady states

Evaluating the Jacobian matrix of (S15) at the steady state yields

$$\frac{\partial \mathbf{F}}{\partial \mathbf{x}}\left(\overline{M},\overline{P}\right) = \begin{pmatrix} -k_4 & 0 \\ k_3 & -k_5 \end{pmatrix}. \tag{S19}$$

(Here, as for the auto-activation model, $\mathbf{x}$ and $\mathbf{F}$ denote the state vector and vector field of (S15) respectively). The eigenvalues of the Jacobian are $-k_4$ and $-k_5$. The steady state $\left(\overline{M},\overline{P}\right)$ is therefore always a stable node, and bifurcations cannot occur [2].

S2.3 Sensitivity-based robustness

Writing $\mathbf{k} = (a, k_1, k_2, k_3, k_4, k_5, k_D, T, N)^T$ for the vector of model parameters, the derivative of the vector field w.r.t. $\mathbf{k}$ is

$$\frac{\partial \mathbf{F}}{\partial \mathbf{k}}([M],[P]) = \begin{pmatrix} \frac{k_1+k_2\left(\frac{T}{k_D}\right)^N}{1+\left(\frac{T}{k_D}\right)^N} & 0 \\ \frac{a}{1+\left(\frac{T}{k_D}\right)^N} & 0 \\ \frac{a\left(\frac{T}{k_D}\right)^N}{1+\left(\frac{T}{k_D}\right)^N} & 0 \\ 0 & [M] \\ -[M] & 0 \\ 0 & -[M] \\ \frac{aN(k_1-k_2)\left(\frac{T}{k_D}\right)^N}{k_D\left(1+\left(\frac{T}{k_D}\right)^N\right)^2} & 0 \\ -\frac{aN(k_1-k_2)\left(\frac{T}{k_D}\right)^{N-1}}{k_D\left(1+\left(\frac{T}{k_D}\right)^N\right)^2} & 0 \\ -\frac{\ln\left(\frac{T}{k_D}\right)a(k_1-k_2)\left(\frac{T}{k_D}\right)^N}{\left(1+\left(\frac{T}{k_D}\right)^N\right)^2} & 0 \end{pmatrix}^T. \tag{S20}$$

At the steady state, this matrix can be written in the form below:

$$\frac{\partial \mathbf{F}}{\partial \mathbf{k}}(\overline{M},\overline{P}) = \begin{pmatrix} \beta k_1 \overline{P} & 0 \\ \frac{a}{1+\gamma^N} & 0 \\ \frac{a\gamma^N}{1+\gamma^N} & 0 \\ 0 & \frac{k_5}{k_3}\overline{P} \\ -\frac{k_5}{k_3}\overline{P} & 0 \\ 0 & -\overline{P} \\ -\frac{ak_1}{k_D}\gamma f'(\gamma) & 0 \\ \frac{ak_1}{k_D}f'(\gamma) & 0 \\ \frac{ak_1}{N}\gamma\ln(\gamma)f'(\gamma) & 0 \end{pmatrix}^T. \tag{S21}$$

A similar analysis to that employed in section S1.3 then yields the required expression for $\frac{\partial \ln \overline{P}}{\partial \ln \mathbf{k}}$:

$$\frac{\partial \ln \overline{P}}{\partial \ln \mathbf{k}} = \begin{pmatrix} 1 & \frac{1}{\beta\overline{P}(1+\gamma^N)} & \frac{K\gamma^N}{\beta\overline{P}(1+\gamma^N)} & 1 & -1 & -1 & -\frac{\gamma f'(\gamma)}{\beta\overline{P}} & \frac{\gamma f'(\gamma)}{\beta\overline{P}} & \frac{\gamma \ln(\gamma) f'(\gamma)}{\beta\overline{P}} \end{pmatrix}.$$

The sensitivity of circuit II is therefore given by

$$S = 4 + \frac{1}{\left(\beta\overline{P}\right)^2}\left(\frac{\left(K\gamma^N\right)^2 + 1}{\left(1+\gamma^N\right)^2} + \left(\gamma f'\left(\gamma\right)\right)^2\left(\ln(\gamma)^2 + 2\right)\right). \tag{S22}$$

Note that as $\overline{P}$ depends only on γ through eqn. (S18), S is solely a function of the parameters β, K, γ and N. Also, for large N, the sigmoidal form of $f\left(x\right)$ means that either $\beta\overline{P} \approx 1$ (γ sufficiently small), or $\beta\overline{P} \approx K$ (γ sufficiently large). Moreover, in the first case $\frac{\left(K\gamma^N\right)^2+1}{\left(1+\gamma^N\right)^2} \approx 1$ and $\gamma f'\left(\gamma\right) \approx N\left(K-1\right)\gamma^N$, while in the second case $\frac{\left(K\gamma^N\right)^2+1}{\left(1+\gamma^N\right)^2} \approx K^2$ and $\gamma f'\left(\gamma\right) \approx \frac{N(K-1)}{\gamma^N}$. Hence, in both cases $\frac{\left(K\gamma^N\right)^2+1}{\left(1+\gamma^N\right)^2} \approx \left(\beta\overline{P}\right)^2$ and $\beta\overline{P} \gg \gamma f'\left(\gamma\right)$, implying that $S \approx 5$. For large values of N, robustness therefore has a weak dependence on β, K and γ (and hence on the steady state protein level $\overline{P}$). In direct analogy with circuit I, this means that it should be possible to promote the evolvability of the circuit while maintaining its robustness through appropriate tuning of the parameters comprising β, K and γ. Indeed, although the genotype-phenotype mapping is predominately characterised by an inverse evolvability/robustness relationship, there are genotypes that break this relationship, and their frequency increases with increasing N.

Similarly to the auto-activation model (circuit I), a necessary condition for the inverse E/R correlation to be broken in circuit II is the predicted large Hill coefficient N, together with high values of the composite parameters β and K. Note also that the mean expression level of $\overline{P}$ is lowest in the region of high robustness and high evolvability; this is consistent with the fact that γ is low across all regions of the G-P mapping, and the prediction from the analysis above that $\overline{P} \approx \frac{1}{\beta}$ for small γ values.

References

[1] H. Kuwahara and O.S. Soyer. Bistability in feedback circuits as a byproduct of evolution of evolvability. *Mol Syst Biol*, 8:564, 2012.

[2] S. Strogatz. *Nonlinear Dynamics And Chaos: With Applications To Physics, Biology, Chemistry, And Engineering (Studies in Nonlinearity).* Westview Press, 1st edition, 2001.

[3] M. van Hoek and P. Hogeweg. The effect of stochasticity on the lac operon: an evolutionary perspective. *PLoS Comput Biol*, 3(6):e111, 2007.

[4] M.J.A. van Hoek and P. Hogeweg. In silico evolved lac operons exhibit bistability for artificial inducers, but not for lactose. *Biophys J*, 91(8):2833–2843, 2006.

S3 Supplementary Tables

Table A: Parameter values for the auto-activation circuit (circuit I) (S1), based on the study of Kuwahara and Soyer [1] (the nominal parameter set used in [1] is shown in bold).

Parameter	Description	Values
a	Transcription scaling factor	[0.6, 0.8, **1.0**, 1.2, 1.4]
k_1	Basal transcription rate	[0.005, 0.01, 0.015, **0.02**, 0.025, 0.03]
k_2	Transcription rate	[0.05, 0.1, 0.15, **0.2**, 0.25, 0.3, 0.35, 0.4]
k_3	Max. translation rate	[0.04, 0.07, **0.1**, 0.13, 0.16, 0.19]
k_4	mRNA degradation rate	[0.04, 0.07, **0.1**, 0.13, 0.16, 0.19]
k_5	Protein degradation rate	[0.001, 0.0015, **0.002**, 0.0025, 0.003]
k_D	Half-activation parameter	[40, 45, **50**, 55, 60, 65]
N	Hill coefficient	[0.0, 5.8] in increments of 0.2
α	Composite parameter, see sec. S1.1	0.2 – 309
K	Composite parameter, see sec. S1.1	1.7 – 80

Table B: Parameter values for the auto-activation circuit (circuit I) (S1), based upon experimentally measured rates from the *lac* operon in *Escherichia coli*.

Parameter	Description	Value (Range)	Unit	Comment
a	Transcription scaling factor	100	mM/min	Estimated
k_1	Basal transcription rate	$10^{-10} - 2 \times 10^{-9}$	mM/min	[3, 4] and refs. therein
k_2	Transcription rate	$10^{-7} - 2 \times 10^{-5}$	mM/min	[3, 4] and refs. therein
k_3	Max. translation rate	$10^{-5} - 0.1$	mM/min	Estimated using [3, 4]
k_4	mRNA degradation rate	$0.1 - 1.5$	1/min	[3, 4] and refs. therein
k_5	Protein degradation rate	$0.002 - 0.018$	1/min	[3, 4] and refs. therein
k_D	Half-activation parameter	$10^{-10} - 10^{-6}$	mM	Estimated using [4, 3]
N	Hill coefficient	$0 - 5.6$	-	Estimated
α	Composite parameter	$2 \times 10^{-6} - 2.7 \times 10^{5}$	-	See sec. S1.1
K	Composite parameter	$100 - 10^{5}$	-	See sec. S1.1

Table C: Upper and lower bounds for the parameters of the simple-activation model (circuit II) (S15).

Parameter	Description	Value (Range)
a	Transcription scaling factor	$0.7 - 1.1$
k_1	Basal transcription rate	$1.7 \times 10^{-17} - 78$
k_2	Transcription rate	$2.8 \times 10^{-5} - 7.7 \times 10^{7}$
k_3	Max. translation rate	$0.1 - 1$
k_4	mRNA degradation rate	$0.1 - 1$
k_5	Protein degradation rate	$0.1 - 1$
k_D	Half-activation parameter	$0.005 - 117$
T	TF concentration	$7 \times 10^{-6} - 0.13$
N	Hill coefficient	$0.5 - 10.5$
β	Composite parameter, see sec. S2.1	$0.1 - 5.7 \times 10^{16}$
K	Composite parameter, see sec. S2.1	$1.2 - 9.9 \times 10^{14}$
γ	Composite parameter, see sec. S2.1	$9.2 \times 10^{-7} - 1.49$

S4 Supplementary Figures

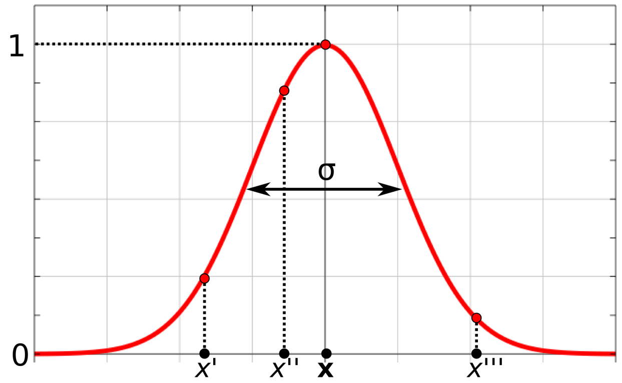

Figure A: Diagram demonstrating the probabilistic robustness measurement principle. The steady state value of a focal genotype $\mathbf{x}$ is taken as the mean of a normal distribution with a chosen value of σ, normalised to have a maximum value of 1. Perturbed steady states $x', x'', x''', \ldots$ resulting from parameter perturbations around the focal genotype, are then mapped onto this normal distribution. This mapping is interpreted as the propensity of a perturbed genotype to maintain the steady state value of the focal genotype. The robustness of the focal genotype is then computed as the mean of all propensity values obtained, and lies in the interval [0,1].

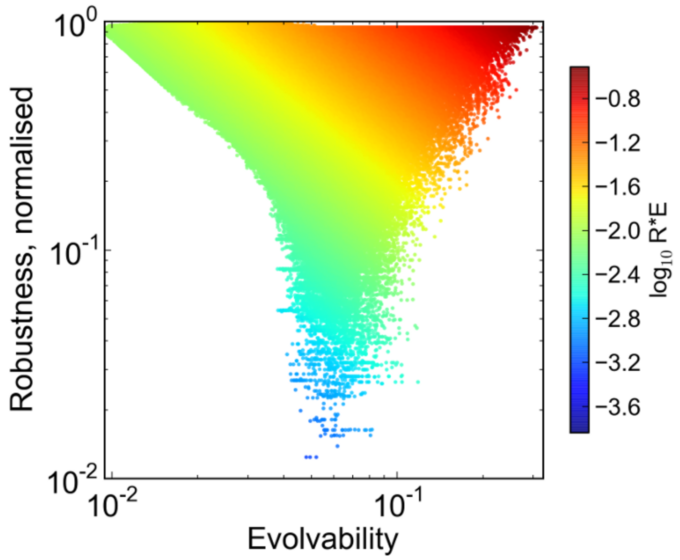

Figure B: The evolvability/robustness relationship for circuit I, colour-coded by the product of robustness and evolvability, $(R \times E)$. Evolvability was calculated using 1-mutant neighbours of each genotype; robustness was calculated using the sensitivity-based measure. High $(R \times E)$ values correspond to the region of the G-P mapping containing genotypes that are both evolvable and robust.

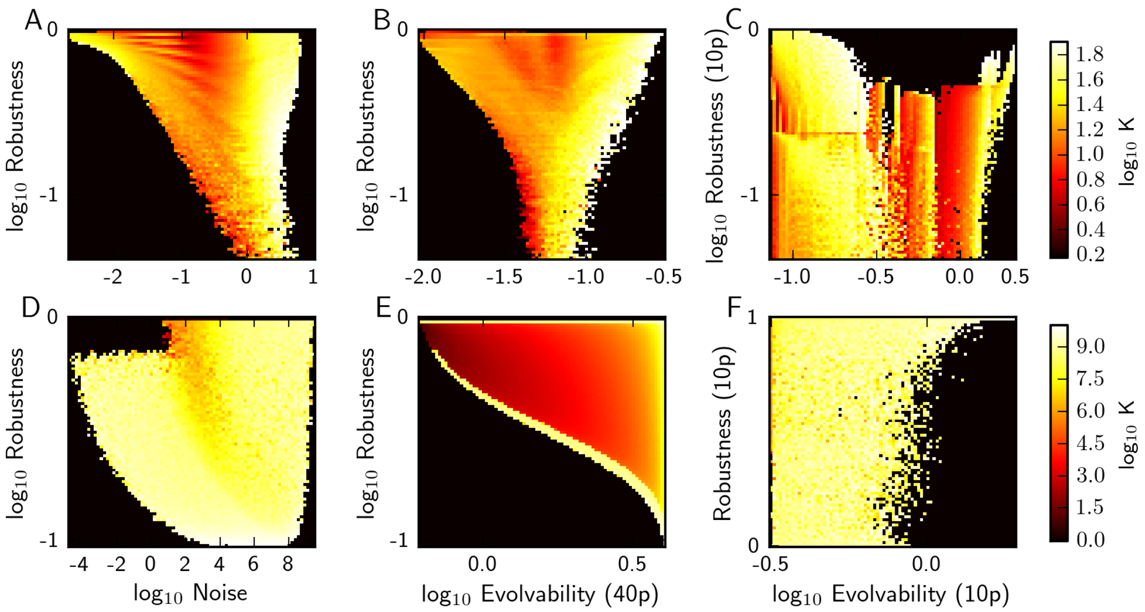

Figure C: Values of the composite parameter K in the G-P mappings of circuit I (A-C) and circuit II (D-F). The combinations of robustness and evolvability measures shown are identical to those used in Figure 2 of the main manuscript. In each plot, brighter colours signify higher mean values of K.

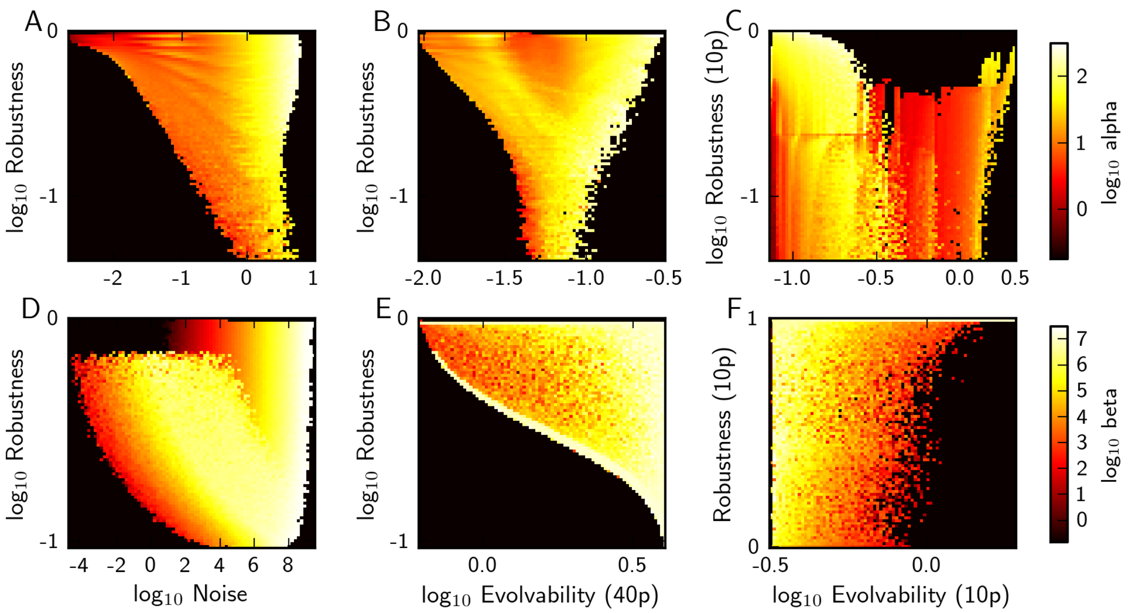

Figure D: A-C: values of the composite parameter α in the G-P mappings of circuit I. D-F: values of the composite parameter β in the G-P mappings of circuit II. The combinations of robustness and evolvability measures shown are identical to those used in Figure 2 of the main manuscript. Brighter colours signify higher mean values of α (A-C) and β (D-F).

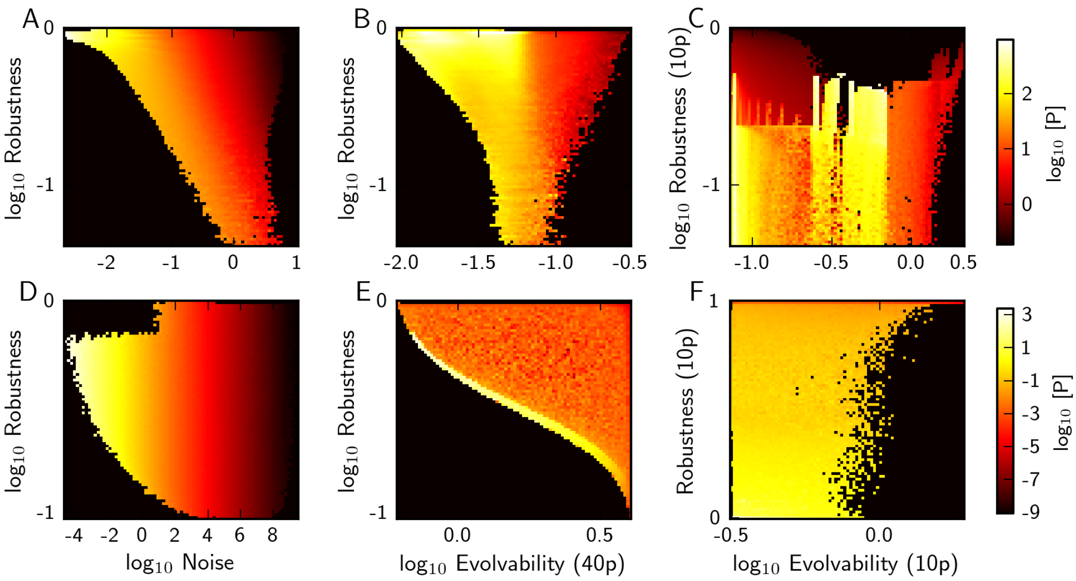

Figure E: Steady state protein expression levels $\overline{P}$ in the G-P mappings of circuit I (A-C) and circuit II (D-F). The combinations of robustness and evolvability measures shown are identical to those used in Figure 2 of the main manuscript. Brighter colours signify higher $\overline{P}$ values in each panel.

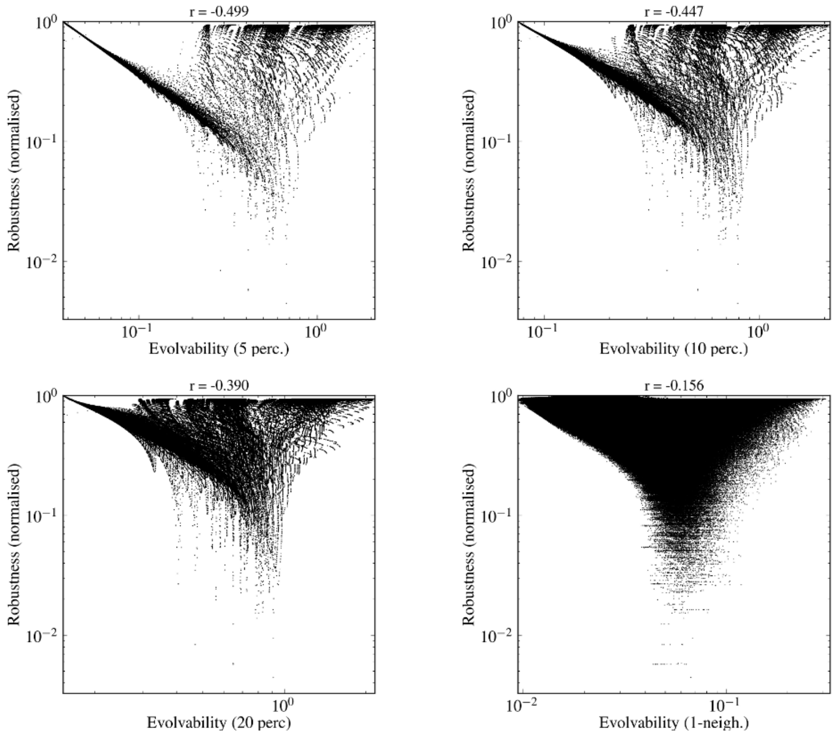

Figure F: The evolvability/robustness relationship for circuit I. Robustness was calculated using the sensitivity-based measure, while evolvability was calculated using: (i) parameter perturbations of $\pm5\%$, $\pm10\%$, $\pm20\%$; and (ii) the 1-mutant neighbours for each genotype. r values indicate the Pearson correlation coefficient in each case.

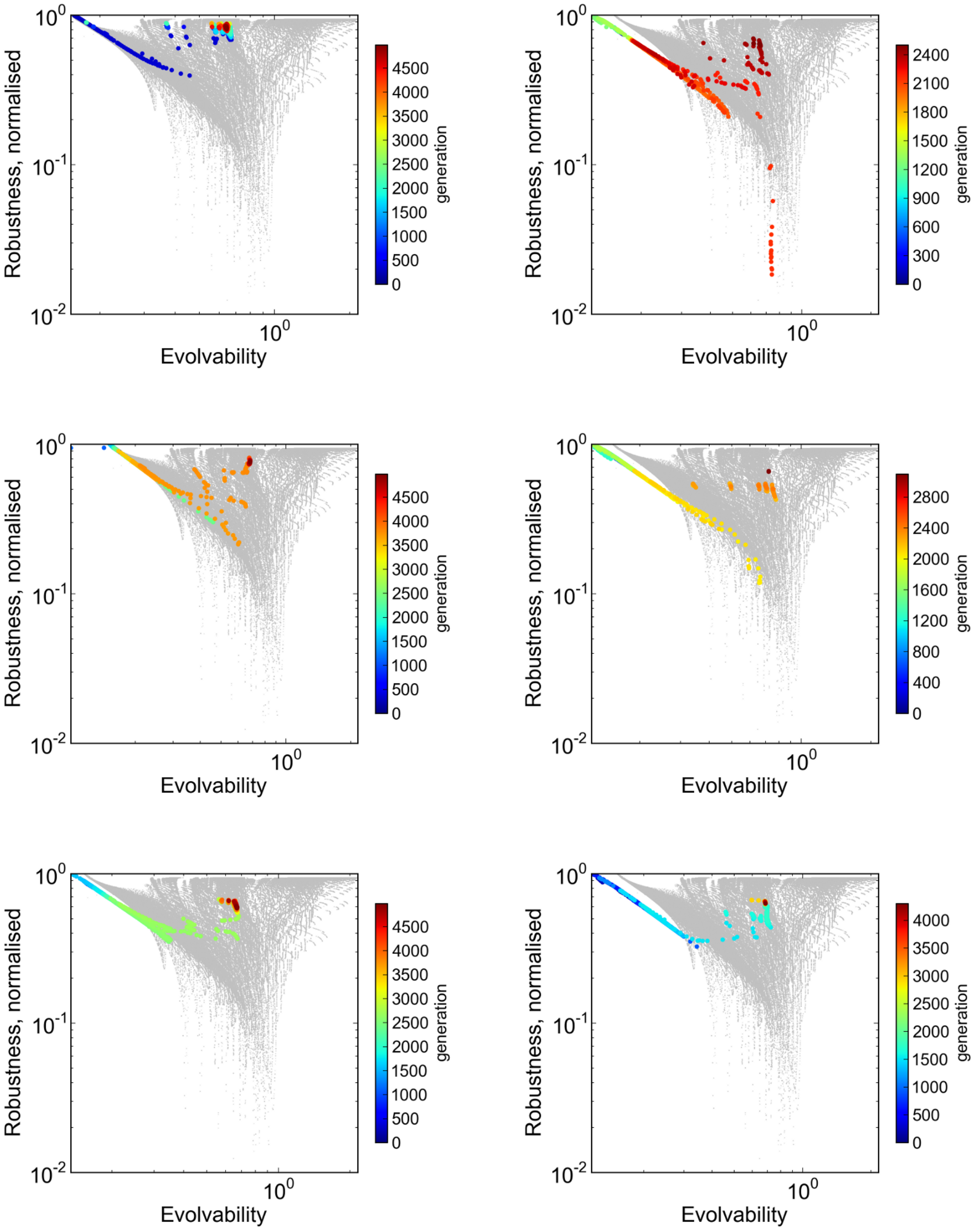

Figure G: The evolvability/robustness relationship for circuit I, as obtained from stochastic simulations in fluctuating environments. The evolvability/robustness relationship for monostable genotypes in the G-P mapping is shown as a grey backdrop, while the mean monostable genotypes arising from the evolutionary simulations are shown in colour (colour levels indicate the generation number in each case). Evolvability was calculated using ±20% parameter fluctuations, while robustness was calculated using the sensitivity-based measure. The results of 6 different evolutionary simulations are shown (cf. Figure 7A of the main paper).

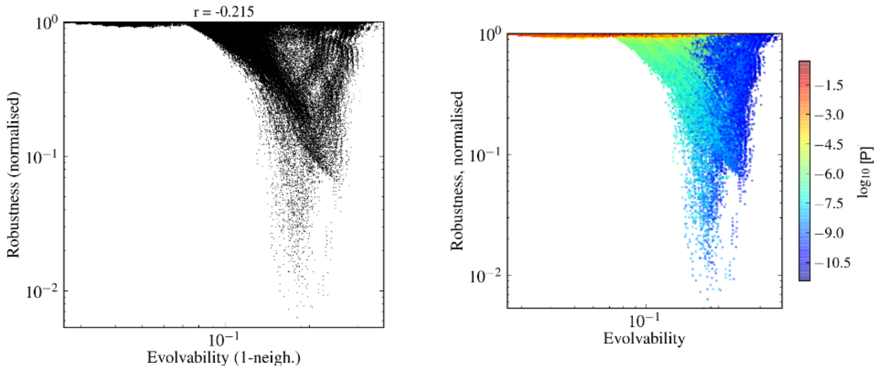

Figure H: The evolvability/robustness relationship for circuit I using parameters from the *lac* operon in *E. coli.* Evolvability was calculated using 1-mutant neighbours; robustness was computed using the sensitivity-based measure. Left panel: all monostable genotypes. Right panel: all monostable genotypes, colour-coded by the steady state protein expression level $\overline{P}$. The r value in the left panel figure indicates the Pearson correlation coefficient.

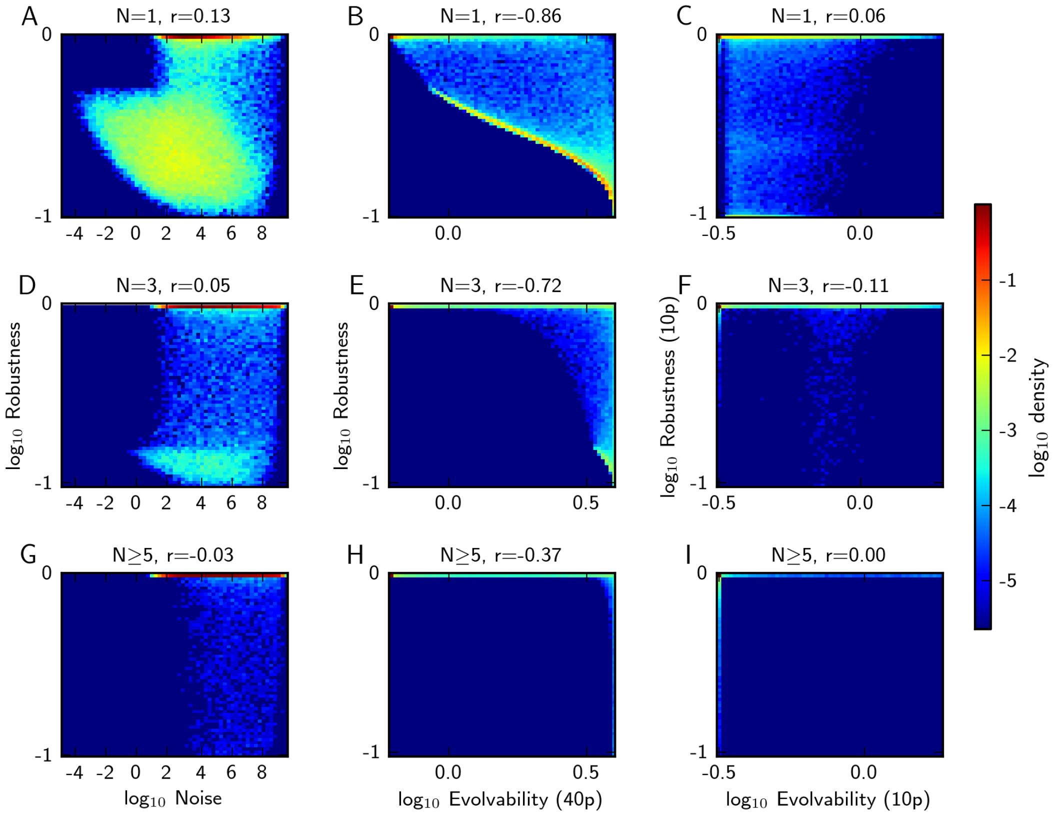

Figure I: The G-P mappings of circuit II for different values of N. The first column (A,D,G) shows the relationship between sensitivity-based robustness and intrinsic noise; the second column (B,E,H) shows sensitivity-based robustness versus evolvability, when evolvability is computed using 40% parameter perturbations; the third column (C,F,I) shows robustness against evolvability when both are computed using 10% parameter perturbations. Colours indicate genotype density and r indicates the Pearson correlation coefficient.

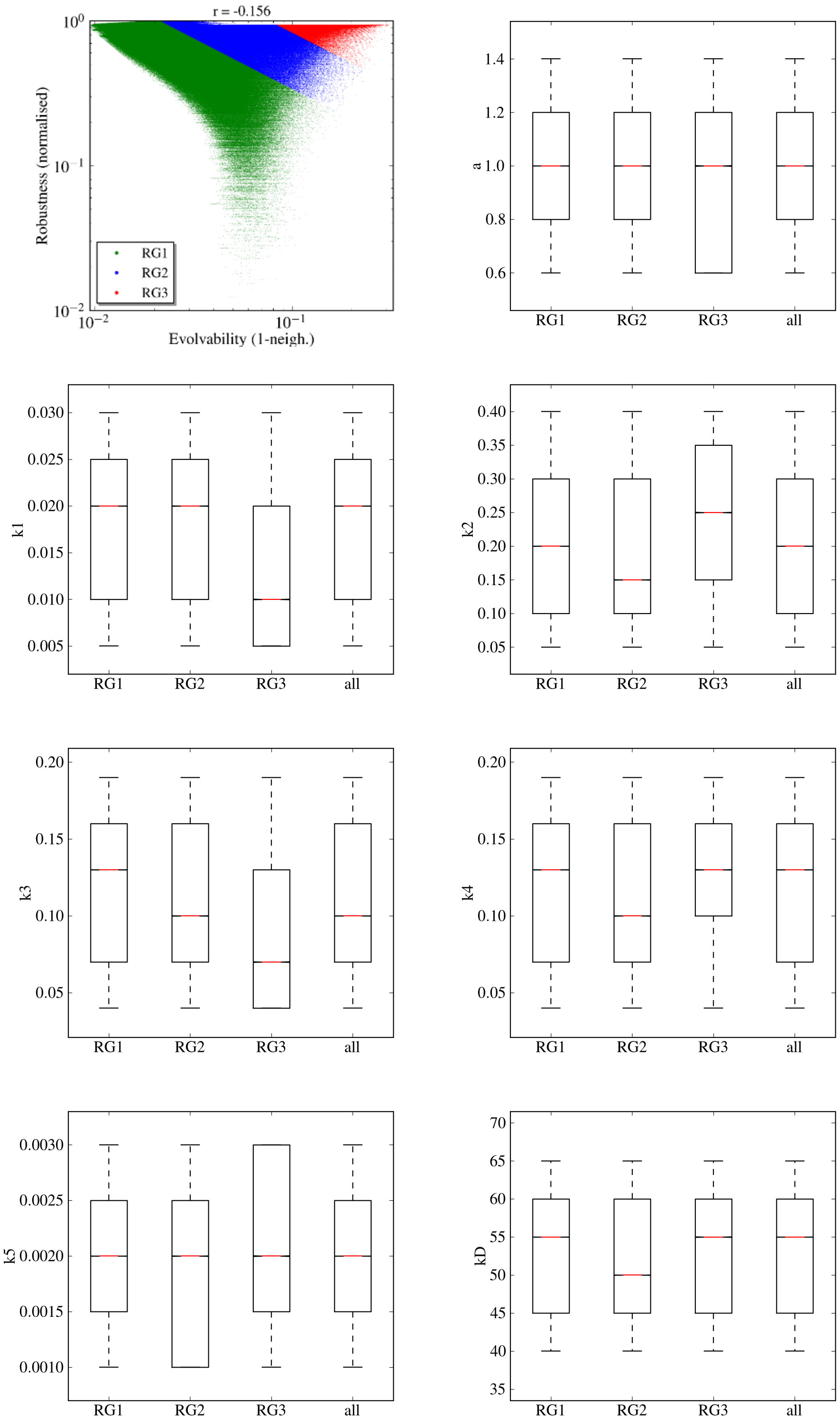

Figure J: The relationship between sensitivity-based robustness and evolvability computed with large-scale parameter perturbations (1-neighbours) for circuit I, shown together with the distributions of individual parameters in regions RG1-RG3 (these are defined according to their distance to the $N = 1$ regression line). The r value in the top left panel indicates the Pearson correlation coefficient.

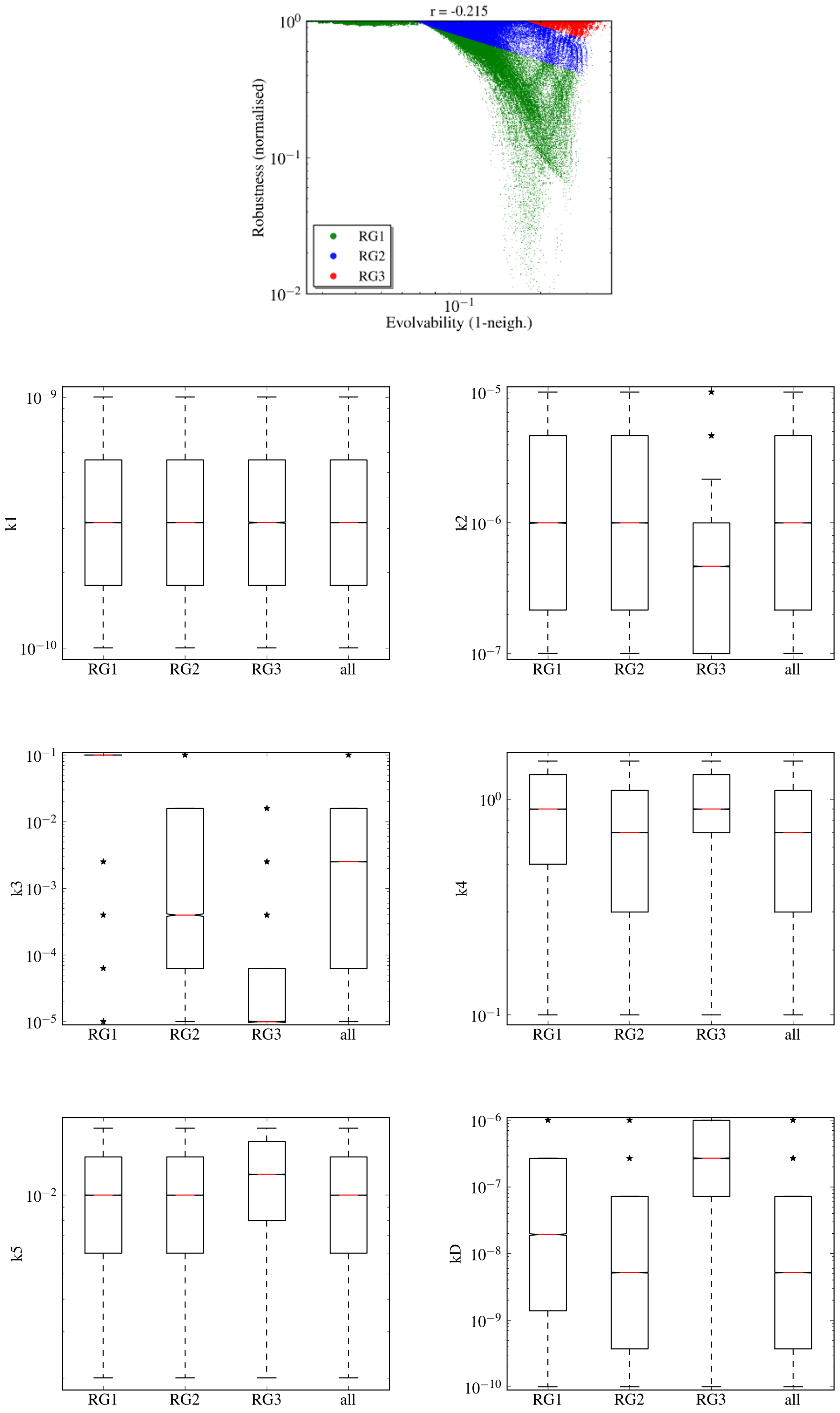

Figure K: The relationship between sensitivity-based robustness and evolvability computed using large-scale perturbations (1-neighbours) for circuit I using parameters from the *E. coli lac* operon, shown together with the distributions of individual parameters in regions RG1-RG3 (these are defined according to their distance to the $N = 1$ regression line). The r value in the top panel indicates the Pearson correlation coefficient.
